# What goes on in digital behaviour change interventions for weight loss maintenance targeting physical activity: A scoping review

**DOI:** 10.1177/20552076221129089

**Published:** 2022-11-06

**Authors:** Jorge Encantado, António L Palmeira, Carolina Silva, Falko F Sniehotta, R James Stubbs, Maria João Gouveia, Pedro J Teixeira, Berit L Heitmann, Marta M Marques

**Affiliations:** 1 Centro Interdisciplinar para o Estudo da Performance Humana (CIPER), Faculdade de Motricidade Humana, 37809 Universidade de Lisboa, Cruz Quebrada, Portugal; 2 APPsyCI – Applied Psychology Research Center Capabilities & Inclusion, ISPA – Instituto Universitário, Lisboa, Portugal; 3 Centro de Investigação em Desporto, Educação Física, Exercício e Saúde (CIDEFES), Universidade Lusófona, Lisboa, Portugal; 4 8809 Trinity College Dublin, ADAPT SFI Research Centre & Trinity Centre for Practice and Healthcare Innovation, College Green, Dublin, Ireland; 5 NIHR Policy Research Unit Behavioural Science, Faculty of Medical Sciences, Institute of Health & Society, Newcastle University, Newcastle, UK; 6 Department of Public Health, Preventive and Social Medicine Center for Preventive Medicine and Digital Health, Heidelberg University, Mannheim Medical Faculty, Mannheim, Germany; 7 School of Psychology, Faculty of Medicine and Health, 4468 University of Leeds, Leeds, UK; 8 Research Unit for Dietary Studies at The Parker Institute 53166 Bispebjerg and Frederiksberg Hospital, part of the Copenhagen University Hospital – The Capital Region, Copenhagen, Denmark; 9 The Department of Public Health, Section for General Medicine, University of Copenhagen, Copenhagen, Denmark; 10 Comprehensive Health Research Centre, NOVA Medical School, Universidade Nova de Lisboa, Lisbon, Portugal

**Keywords:** Health behaviour, weight loss maintenance, physical activity, exercise, digital technology, review

## Abstract

**Objective:**

To identify the core components of digital behaviour change interventions for weight loss maintenance targeting physical activity, in terms of: (i) behaviour change techniques, (ii) mechanisms of action, (iii) modes of delivery, (iv) dose and (v) tailoring/personalization. In addition, the links between these components were investigated.

**Methods:**

A literature search was performed in five electronic databases: PubMed, Embase, CINHAL, PsycINFO and Web of Science. Two reviewers independently screened the identified articles and extracted data related with the study characteristics and behaviour change techniques, mechanism of action, mode of delivery, dose, and tailoring, using standardized classifications whenever available (e.g. behaviour change techniques taxonomy).

**Results:**

Seventeen articles reporting 11 original studies were selected. Two studies were protocols, 9 studies presented results for weight change and all but one showed no significant differences between the intervention and control groups. Eight studies (73%) provided adequate information on behaviour change techniques. Five studies (45%) provided partial information about how the behaviour change techniques were linked to mechanisms of action, and only one study (0.9%) described these links for all the techniques. Around half of the studies reported the modes through which behaviour change techniques were delivered. Descriptions of dose were present in most studies, but with minimal information. The use of tailoring or personalization approaches was mentioned in eight studies (73%), but descriptions of what was tailored and how were minimal.

**Conclusions:**

The compilation of information regarding intervention components was difficult due to the lack of information and systematization in reporting across papers. This is particularly true for the reporting of the links between behaviour change techniques and the other core intervention components. This information is crucial to help us understand in the context of behaviour change interventions what works or does not work, how it works and why.

## Introduction

Obesity is related to an increased risk of development of major non-communicable diseases, namely cardiovascular diseases, type 2 diabetes and some types of cancers.^1,2^ A meta-analysis of worldwide weight control attempts (72 studies; n  =  1,189,942) showed that 42% of adults from the general population and 44% from ethnic minority populations are trying to lose weight, and 23% reported trying to maintain their weight annually.^3^

While behavioural interventions have shown beneficial effects in the short term in reducing weight, most people experience longer-term weight regain, hindering the health benefits associated with weight loss.^4,5^ It is therefore crucial to implement behavioural interventions that successfully promote weight loss maintenance (WLM), that is, intentional loss of weight of at least 10% of one's body weight that is successfully kept off for at least one year.^6^ Besides healthy dietary behaviours, a core behaviour to be targeted in WLM interventions is physical activity (PA).^7,8^ It is documented that for every additional 10 min of total PA, there is a decrease in the risk of weight management lapses by 1%.^9^ Further, physically active individuals (> 60 min of daily PA) with overweight seeking weight loss have a lower risk of lapse (5%) when compared with inactive individuals (12% risk).^9^ Recent reviews focusing on the strategies used by successful weight loss maintainers highlighted the beneficial role of PA.^3,10^

Digital behaviour change interventions (DBCIs), defined as coordinated sets of activities or products designed to change specified behavioural patterns (e.g. PA) of an individual, group or population through digital technology such as mobile apps, wearable technology (e.g. activity trackers) or websites,^11^ are a viable option for WLM as they have great potential to improve efficiency of behavioural interventions in the long term, with high reach, precision and scalability.^12^

The literature of weight loss is extensive and findings on DBCIs are promising (e.g. ^13–17^). However, in the context of WLM, there are fewer reviews and most report small effects of DBCIs with considerable between-study variability. In a recent review of systematic reviews,^18^ web-based interventions for weight loss and WLM were more effective than minimal or control conditions, but results were inconsistent across reviews that compared with non-web-based interventions. Contrastingly, in the Hutchesson's and colleagues^19^ systematic review including seven DBCIs for WLM, six studies (86%) did not find significant differences on pre- and post-intervention weight change between DBCIs and control (self-directed, no intervention, written materials or general health related messages). In another review by Beleigoli and colleagues^14^ including 11 studies, sensitivity analysis showed that there was greater weight loss in the web-based intervention groups in comparison with non-technology intervention group in studies with less than 6 months follow-up (MD −2.13; 95% CI: −.2.71 to −1.55), but no differences were found when follow-up exceeded 6 months (MD −1.7; 95% CI: −2.1 to 1.76).

To be able to develop and implement effective DBCIs for WLM, we need to identify what are the components included in these interventions and what is the evidence and the theoretical underpinnings of the choices made in the selection of these components. By components of behaviour change interventions, we are referring to: (i) the intervention content, that is, the behaviour change techniques (BCT) that are implemented (e.g. goal setting),^20^ (ii) the theoretical principles that contextualize selected BCTs (e.g. self-efficacy), that is, that are hypothesized to be the means by which given BCT influences behaviour,^21^ (iii) their mode of delivery (MoD), that is, the way(s) through which the interventions are delivered (e.g. using an app),^22^ (iv) if the DBCI content is selected or modified based on characteristics of the population or context, that is, their tailoring or personalization features,^23^ (v) and the intensity with which the DBCI is being delivered.^24^ A key element for replication and accumulation of evidence, is to have access to research studies that map these different components both conceptually and empirically so that one can understand the rationale for choosing a given MoD for a given BCT and make informed choices in future trials.

The development of standardized classifications and descriptions of intervention components, through taxonomies and ontologies, facilitate the identification, description and reporting of the components of behaviour change interventions, facilitating comparison and accumulation of evidence. The Behaviour Change Techniques Taxonomy v1 is probably the best-known example^20^ of a classification system describing the content of behaviour change interventions. Building on this work, the Human Behaviour-Change Project,^11,23^ proposed to develop a Behaviour Change Intervention Ontology to classify and organize other components of behaviour change interventions (such as the setting, delivery and behaviours), with the aim of creating automated evidence searching, synthesis and interpretation to answer questions that are variants of ‘What works, compared with what, how well, with what exposure, with what behaviours (for how long), for whom, in what settings and why?’

To the best of our knowledge, systematic reviews of DBCIs targeting PA in WLM contexts^13–19,25–27^ mainly report the efficacy of the included trials in changing the target behaviour and outcome. These reviews were not aimed at describing the components of behaviour change interventions systematically and in detail, except for BCTs. Further, they do not provide an overarching mapping of the links between the core components of the interventions. This approach can inform us about the current state of evidence and knowledge gaps in DBCIs targeting PA in WLM contexts.

## Purpose of the scoping review

The aim of this scoping review was to identify and describe the core components of DBCIs for WLM targeting PA, that is, digital technology platforms such as mobile apps, mobile and wearable technology (e.g. cell phones, activity trackers) that coordinate a set of activities designed to change specified behavioural patterns in PA. Specifically, the objectives of the present review were to:

1. Identify the content of these interventions (i.e. which BCTs have been reported to be delivered), and how this content was reported to be delivered (MoD, dose and tailoring).
2. Identify the hypothesized theoretical principles (mechanisms of action (MoA)) that support the choices for the content and delivery of the DBCIs.
3. Examine the links between these components that are reported in the literature and how these are described, that is if based on conceptual approaches and/or evidence.

## Methods

This review is reported in accordance with the PRISMA extension for Scoping Reviews guidelines (PRISMA-ScR).^28^ The checklist and the protocol are both publicly available in the Open Science Framework repository (OSF Repository Files). The current scoping review is consistent with Arksey and O’Malley^29^ guidelines by (i) identifying the research question, (ii) identifying relevant studies, (iii) selecting studies in a systematic manner based on the inclusion criteria, (iv) charting the data integrating numerical summary and qualitative thematic synthesis based on existing taxonomies and ontologies for DBCIs and (v) collating and reporting a narrative account of findings in two ways: first for each component, and next, for the links between each component and the BCT.

### Search strategy

A comprehensive literature search was conducted in five electronic databases: PubMed; Embase; CINHAL; PsycINFO and Web of Science. Two reviewers (JE, CCS) independently screened the identified articles according to the predefined inclusion and exclusion criteria. In addition, we performed a manual cross-referencing of bibliographies cited in previous reviews.^14,25,26,30^ The search strategy included terms related with (i) the *concept* (DBCIs designed to support WLM, defined as interventions containing at least one component delivered via the internet) and (ii) the *context* (DBCIs for WLM targeting PA). The initial search was conducted in October 2019 and was updated in January 2021 (search terms and information on excluded studies are available in Supplemental File 1).

### Eligibility criteria

Protocols and reports of intervention studies published in English and using any type of experimental designs were selected for this review. There were no restrictions with respect to the length of the intervention and assessment time point(s). Interventions had to explicitly report: (i) using a digital or web-based component to deliver the intervention; (ii) being aimed at WLM; (iii) targeting PA as a main behavioural target; (iv) include samples of adults (≥ 18 years old) who had lost weight prior to entering the study, that is, adults in WLM process. Studies that included pregnant or post-partum women, individuals with eating disorders, major depressive or anxiety disorders, as well as individuals participating in interventions including surgical or pharmacological components were excluded.

### Data charting process

Data was extracted and chartered independently by two researchers for all information for all articles included (JE, CCS). For most components, we used forms based on the guidelines for coding from the respective published taxonomies (BCTTv1), ontologies (MoD) and descriptions (MoAs, Dose). For tailoring and study characteristics we developed new forms. All of these were tested with three papers reporting two trials and calibrated before being applied to the remaining trials. Data was then verified by both researchers and when it was found incomplete for one entity in one researcher's charting table, both researchers verified this incompleteness on the respective article and updated the table to ensure that no information was missing. The inter-rater reliability (whether two researchers capture the same information from a paper) was measured through the percentage of agreement of instances where both researchers had annotated a component. Disagreements were decided through discussion and with the help of a third author (MM) when needed. Finally, all verified extracted information was organized and coded in a final charting table.

The information extracted was inserted in an excel format table with the indication of the exact part of the paper where the information could be found. All articles were assembled by study using the trial name and/or trial reference. The study characteristics extracted for each study were: (i) bibliographic information (authors, year of publication, and reference), (ii) country where the study took place, (iii) sample characteristics (sample size, gender, age, body mass index), (iv) study design, (v) intervention characteristics (name, aim, length of intervention and follow-up) and (vi) results for the outcomes of interest (weight, PA).

Further, the following characteristics were coded for its presence/absence:

- BCTs: Labels and descriptions of BCTs were identified and extracted from the included studies and then coded using the Behaviour Change Techniques Taxonomy (v1) coding guidance.^20^ This taxonomy provides an extensive list of 93 clearly labelled, well-defined BCTs, clustered into 16 exclusive groupings.
- MoA: Information on the theoretical principles were extracted using the list of MoA from Carey and colleagues.^21^ This list includes 26 MoA taken from the 14 theoretical domains as described in the Theoretical Domains Framework^31^ and the 12 most frequently occurring mechanisms derived from a set of 83 behaviour change theories.^32^
- MoD: Information about MoDs were extracted using the MoD Ontology v1 developed by Marques et al.^22^ This ontology presents a four-level hierarchical structure comprising 65 modes of delivery, organized by 15 upper-level classes: Informational, Environmental change, Somatic, Somatic alteration, Individual-based vs Pair-based vs Group-based, Uni-directional vs Interactional, Synchronous vs Asynchronous, Push vs Pull, Gamification, Arts feature.
- Dose: Information about dose was extracted based on the elements of intensity (what is the intensity with which the intervention is being delivered) described by Dombrowski et al.^24^: duration of intervention, number of contacts, length of contacts, frequency, spacing (e.g. constant, variable) and sequencing of BCTs.
- Tailoring: In this scoping review, we adopted a broad definition of tailoring from the Behaviour Change Intervention Ontology^23^ that can capture other tailoring-related features such as personalization and individualization – *the process by which a behaviour intervention is selected or modified based on characteristics of the population or context.*^23^ For each study we coded, (i) if the intervention used any features of tailoring, and (ii) the description of the tailoring process.

First, information about overall use of BCTs, MoAs, MoD, Dose, and Tailoring components were extracted regarding the intervention conditions. Second, each BCT description was specifically screened and classified as including or not including information about the links with each component: MoAs, MoD, Dose, and Tailoring.

Information on extracted intervention characteristics was summarized and is presented in Supplemental Tables and Figures: Summary (Supplemental File 2), MoD (Supplemental File 3), and Dose (Supplemental File 4). Due to the methodological nature of the present review of mapping key constructs and their relationships and identify gaps in existing research, a qualitative approach was used to categorize the information in each category originated from the selected taxonomies and ontologies.^20,22–24^ When suitable, a quantitative summary is presented using descriptive statistics. A ‘mapping’ overview table was developed to summarize conclusions from the extracted information and to highlight the relationships between concepts of analysis (BCTs, MoAs, MoDs, Dose and Tailoring). This approach was used to adequately summarize the intervention design decisions. The following criteria were used: the description of each BCT was extracted (Supplemental File 5) and analyzed (Supplemental File 6) in terms of its implementation (reported or not); the link with MoA (reported or not); the link with MoD (reported or not); the link with Dose (reported or not); Tailoring components (if it was used or not). For each study we calculated the overall prevalence for each category based on BCTs descriptions.

## Results

### Study characteristics

**Figure 1.**
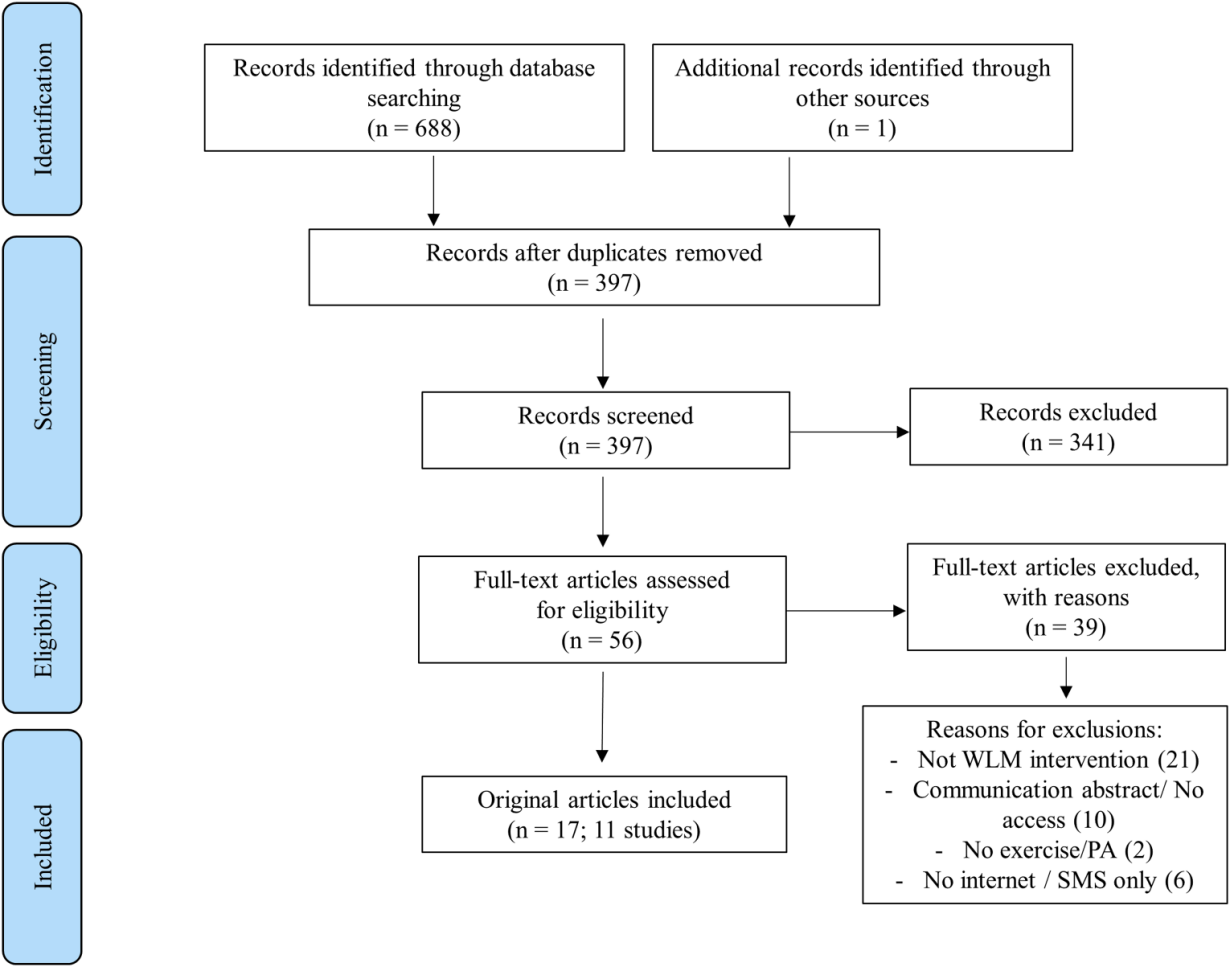
Flow diagram search strategy.

The literature search yielded a total of 688 articles (Pubmed *n*  =  64; Web of Science *n*  =  242; PsycInfo *n*  =  76; Embase *n*  =  247; CINHAL *n*  =  59). Manual search returned one additional article. Duplicates were removed, and abstracts were screened based on the predefined criteria of inclusion. Fifty-six articles were kept for full-text screening and 17 articles reporting on 11 studies were selected for this review (Figure 1).

Study characteristics are summarized in Table 1. The information regarding interrater agreement was found to be good (>80% agreement) for most of the categories in all components (143/137; 96% of the overall components). The categories with the lowest agreement were in Dose component: Regularity (36% agreement) and Number of Contacts (45% agreement) (details are available in Supplemental File 1). From the selected articles, 7 were study protocols and 10 were intervention reports (i.e. reporting main effect or secondary analysis). One study was reported in four articles.^33–36^ Two trials had only the protocol paper published at the moment of the submission of the current review.^37,38^ All studies were randomized controlled trials (RCTs) of various types (i.e. parallel RCTs, factorial, etc.). About one-third of the studies (4/11; 36%) were part of larger trials containing an initial weight loss program. Most studies were conducted in English-speaking countries (9/11, 82%). Sample size ranged from 49^39^ to 880^35^ participants (median  =  95). Duration of the studies ranged from 3 months^40^ to 30 months^35^ (median  =  12). Results concerning weight change were reported in 9 of the 11 studies, the majority of which showed no significant differences between the intervention and control groups. The exception was the trial by Thomas and colleagues,^39^ in which the intervention group maintained an average weight loss of 10%, significantly greater than the comparative group. PA outcomes were reported in six interventions, of these three reported no significant differences between groups,^41–43^ two observed a decrease in PA in the group receiving DBCI in comparison to those receiving personal contact,^35,44^ and one study found a small significant difference in which the intervention group was significantly more physically active than the control group.^45,46^ All studies focused on PA and dietary behaviour except Nakata et al.^43^ that intervened on PA only. Four studies specifically targeted increments in step-count,^39,44,47,48^ four studies targeted general physical,^38,39,43,49^ three studies targeted exercise but did not specify the intensity,^40,45,49^ and two studies reported targeting moderate to vigorous PA.^41,44,48^

**Table 1.** Study characteristics.

Authors, year	Country	Name/Reference of the program	Design (type, arms)	Intervention	Sample (N, Age M(SD), BMI, % women)	Length of intervention (follow-up)
Brindal et al., 2016; Brindal et al., 2019	Australia	Motimate	Parallel RCT (1 IG, 1 CG)	Mobile app (Motimate) to assist in maintenance of weight loss.	N = 88 Age = 45.1 (13.9) BMI = 20.9 to 60.8 75% women	3 months (3 months)
Collins et al., 2010; Collins et al., 2017	Australia	CTRN12610000197033	RCT (1 IG, 1 CG)	Internet-based intervention for weight maintenance.	N = 227 Age = 42.3 (10.1) BMI = 30.4 (4.1) 56% women	12 months
Coughlin et al., 2013; Brantley et al., 2008; Funk et al., 2010; Stevens et al., 2008	United States of America	WLM Website BFS	RCT (2 IG, 1 CG)	Internet-based intervention for weight loss maintenance.	N = 880 Age = 55.9 (8.7) BMI = 30.9 (4.7) 35.7% women	30 months
Espel-Huynh et al., 2019	United States of America	Refresher Weight Loss	Pragmatic type 2 effectiveness - implementation hybrid design (2 IG, 1 CG)	An interactive, online weight loss program for weight loss maintenance.	NA	12 months
Evans et al., 2015; Sniehotta et al., 2019	United Kingdom	NuLevel	Randomized superiority trial (1 IG, 1 CG)	Mobile internet platforms and technology assisted program for weight loss maintenance.	N = 288 Age = 41.8 (11.5) BMI = 30.9 77.5% women	12 months
Gerber et al., 2013	United States of America	Exercise Ur Faith	RCT (1 IG, 1 CG)	A telehealth weight maintenance programme.	N = 88 Age = 50(8) BMI = 32.4 100% women	9 months
Leahey et al., 2016	United States of America	Providence	RCT (2 IG, 1 CG)	Internet-based weight loss maintenance intervention.	N = 75 Age = 48.5 (10.7) BMI = 31.5 (5.8) 85.3% women	10 months
Nakata et al., 2019	Japan	UMIN	RCT (1 IG, 1 CG)	Internet-based intervention to promote weight loss maintenance.	N = 95 Age = 55.9 (6.1) BMI = 28.3 (2.8) 62.1% women	24 months
Scott et al., 2019	Denmark, Portugal, United Kingdom	NoHoW	2 × 2 RCT (3 IG, 1 CG)	Digital toolkit using a mobile-enabled website for weight loss maintenance.	NA	6 months (12 months)
Thomas et al., 2011	United Kingdom	Weight Loss Clinics	RCT (1 IG, 1 CG)	Regular e-mail messages to support weight loss maintenance.	N = 49 Age = 44.7 (13.6) BMI = 32.9 (10) NR % women	6 months
Wing et al., 2008	United States of America	Stop Regain	RCT (2 IG, 1 CG)	Internet-based intervention for weight loss maintenance.	N = 261 51.2 (10.1) 28.5 (4.8) 82% women	18 months

Note: Age M: age mean; BMI: body mass index; CG: control group; IG: intervention groups; NA: not applicable; RCT: randomized controlled trial.

### Behaviour change techniques

All studies reported the use of behavioural techniques (full details available in Supplemental File 5), although only three^38,40,46^ reported using the BCTTv1^20^ to classify them. BCTs were described adequately in eight studies (8/11; 73%) whereas three studies did it for some BCTs but not for all. Overall, 36 BCTs were used across the studies. Some interventions implemented the same BCT multiple times, using more than one MoD (e.g. participants instructed to self-monitor outcomes of behaviour during a *face-to-face* contact or via *email*) or for different behaviours and/or health outcomes (e.g. implementing goal setting for *weight* or for *expended calories*). The study that reported using more BCTs was NuLevel,^46^ 10 times more (*n*  =  30) than the study with the least number of BCTs reported (*n*  =  3).^38^ On average 12 BCTs were reported per study. The most frequently reported BCTs were *self-monitoring of outcome(s) of behaviour* (*n*  =  9; 82%) and (*n*  =  8; 73%). The BCTs *goal setting of behaviour* (PA), *self-monitoring of behaviour* (PA), *feedback on outcome of behaviour* (weight), and *social support (unspecified)* were reported in seven studies (64%). *Problem solving* and *feedback on behaviour* (PA) were reported in six studies (55%). The remaining BCTs were dispersed across studies.

### Mechanisms of action

Overall, 20 hypothesized MoAs were reported across 7 studies (7/11; 64%). The most frequently reported was *Behavioural Regulation* (7/11; 64%) defined as the behavioural, cognitive and/or emotional skills for managing or changing behaviour,^21^ followed by *Social Influences* (i.e. interpersonal processes that can cause oneself to change one's thoughts, feelings or behaviours) (5/11; 45%), and then by *Skills,* and *Environmental Context and Resources* each one reported in four studies (4/11; 36%). The study with more theoretical principles reported was NuLevel^45^ with 15, followed by Motimate^40^ with 12. Next, with smaller numbers, the study by Scott and colleagues^38^ reported three MoAs and Wing and colleagues^44^ reported only one. MoAs were not reported in four studies (4/11; 36%).^37,39,42,43^

### Modes of delivery

Only one study was entirely digital and automated.^38^ The other 10 studies also included non-automatic *distant human interaction* and four of these reported additional *face-to-face interaction* for delivering intervention content.^35,39,46,47^ Most studies used mobile devices or desktop computers as a mean to deliver the intervention content (*n*  =  9 desktop enabled; *n*  =  1 mobile first [39]). The exception was the study by Gerber and colleagues,^42^ which used Home TV videos (DVR) to deliver the intervention content. The most frequently used form of communication was email (9/11; 82%), followed by audio calls or messages (7/11, 64%). Video call/messages were used in three studies (e.g. 1-min videos with intervention content) (3/11; 27%)^37,38,42^ and text/instant messages were used in three others (3/11; 27%).^40,45,48,50^ Only three studies provided wearable technology to monitor PA, that is activity monitors (3/11; 27%^38,43–45^), and four studies provided wireless weighing scales (4/11; 36%^38,43–45^). All but one study also provided a weighing scale to the control group.^43^ Printed materials were included in two studies.^44,45^ All interventions were delivered individually. In addition, one intervention included dyadic communication (i.e. peer support)^47^ and two others in person group sessions.^35,47^ All studies used one-way interactions and mostly using asynchronous communication (e.g. email). Additionally, more than two-thirds used synchronous and reciprocal interactions (e.g. phone calls) (8/11; 73%). Most of the reviewed studies used push message (9/11; 82%) or pull message (9/11; 82%), either to communicate with the participants or to deliver reminders or behavioural prompts to report data, or to adhere to behavioural monitoring. Regarding the format of the content delivered, all studies used written content, six studies reported also using audio content (6/11; 55%), and three studies included video content (3/11; 27%). Images or visual elements were used by 55% of the included studies (6/11), usually in the form of self-monitoring graphs. None of the interventions used gamification features. For full details please refer to Supplemental File 3.

### Dose

Information about the duration of the contacts and interactions was detailed in five studies (5/11; 45%).^35,42,43,45,47^ The order of presentation of intervention content was reported in two studies (2/11; 18%)^37,45^ (full details are in Supplemental File 4). The duration of delivery of each BCT was only reported in the NuLevel trial (10 min for each module of intervention).^45^ The frequency of delivery ranged from daily (4/11; 36%), weekly (5/11; 45%), and monthly contacts (5/11; 45%).

Overall, the number of contacts was poorly reported, or insufficient details were provided. The ‘WLM Website BFS’ intervention^35^ reported one face-to-face orientation session and one reorientation group session at the 12th month; the ‘Motimate’ intervention^40^ reported to have five clinic visits over the period of 24 weeks; the NoHoW trial^38^ was reported to have 26 automatically delivered emails; the study by Nakata and colleagues^43^ reported 24 monthly personalized text messages; and Thomas and colleagues^39^ reported that the Weight Loss Clinics study had 26 email contacts with each participant plus six emails asking weight.

### Tailoring

The use of tailoring was mentioned in eight studies (8/11; 73%), but descriptions of what was tailored and how it was tailored were minimal. Some studies used automated tailored feedback from reports or data retrieved from sensors, others provided tailored content based on weight related changes (e.g. traffic light system that triggers personalized content based on weight variation).

### Links between behaviour change techniques and other intervention components

**Figure 2.**
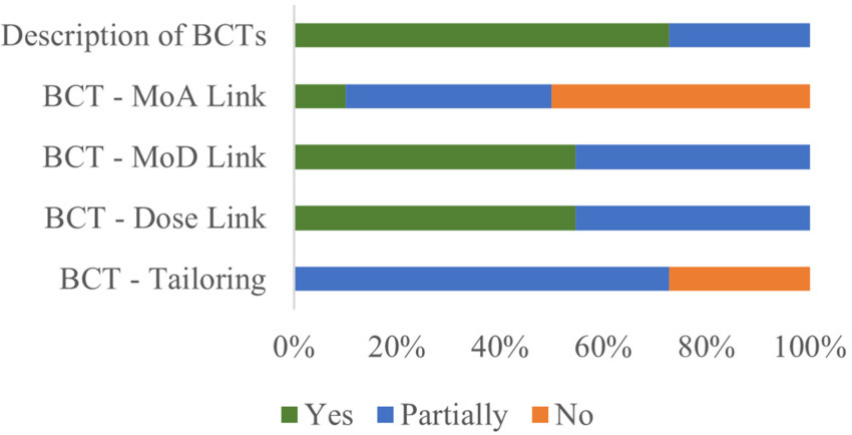
Percentage of components reported based on BCTs’ descriptions.

Most of the studies reported to some extent the information on how BCTs were implemented (i.e. how they were translated into functionalities and content presented to users), what were the hypothesized theoretical principles, how they were delivered (MoD), and with what dose (Figure 2; for full details see Supplemental File 6). However, a single study described the links between all BCTs and theoretical principles,^45^ four studies described it partially, but from these, two studies reported links for only a third of the used BCTs. This means that less than half of the studies reported some information about the link between BCTs and theoretical principles (5/11, 45%).

Even though the language used was not standardized across papers, we identified that 55% (6/11) of the studies described the MoD for each BCT. However, the other five remaining studies did not describe it for all reported BCTs (Figure 2; for full information for each BCT please refer to Supplemental File 6). Frequency of delivery for all BCTs was reported in six studies (6/11; 55%) (e.g. weekly, daily). Lastly, for tailoring, no study fully described the tailoring procedures for all BCTs and the study with the best description considering all used BCTs was the NuLevel.^45^

## Discussion

This scoping review identified the various components used in DBCIs for WLM targeting PA. By systematically extracting and classifying the intervention components from identified studies, we aimed to provide an overview on the current state of DBCIs reporting, identify gaps in the literature and provide new insights on the need to use formal ways of reporting intervention components to facilitate accumulation of evidence and inform future intervention development.

Half of the reported BCTs were reported on two studies. On the other hand, a small number of the 36 BCTs were considered essential to be implemented in DBCIs for WLM, for example, the *self-monitoring of outcomes of behaviour* BCT was used in 82% of the studies reviewed and the *prompts/cues* in 73%. Overall, there is a trend to favour the use of BCTs that are related to the Self-Regulation Theory, such as *goal setting of behaviour*, *self-monitoring of behaviour* and *feedback on outcome of behaviour* (included in 64% of the studies). This is consistent with the literature as self-monitoring is frequently associated with better weight management.^49,51^ However, most studies did not provide monitoring technology and relied on self-reported data to monitor behaviours, define goals, and to provide feedback to participants. It is important to note that some BCTs in the context of weight management and PA would benefit greatly from the use of monitoring technology in terms of its automaticity and easiness, for example to self-monitor the behaviour (e.g. PA) or provide feedback on outcome of behaviour (e.g. weight).

Seven studies fully reported theoretical principles on intervention design but two just identified the underlying theory and did not provide further information on the hypothesized MoAs targeted by the intervention. Indeed, only five studies provided specific information about the links between the hypothesized MoAs and BCTs, that is, provided some extent of information on which MoAs were hypothesized to be impacted by the selected BCTs. Even though these relationships BCTs-MoAs are identified as fundamental to inform the intervention design and to understand why an intervention is effective in achieving the behavioural change targets, only a single study^45^ provided proper detail on this.

It seems that the technological capabilities of DBCIs are not being used to its full potential as the studies relied heavily on static written content. Only six studies took advantage of audio features and only three used videos to deliver intervention content. This might be a result of the limits of the existing technology at the time of intervention development (about half the studies were published before 2015). There is great potential of the technological features available through digital platforms that allow enhanced user engagement and increased effectiveness in delivering complex and dynamic intervention techniques (e.g. individual, and contextual adaptation and tailoring). Optimization designs such as micro-randomized trials may contribute to harness all the potential of DBCIs by providing the means to test more granular aspects of each component of the intervention and further help to refine behaviour change theories and assigned intervention content.^52^

Furthermore, papers reviewed did not systematize the information on the dose of the intervention. Few studies reported systematically the information about the number of modules or materials, and there were limited descriptions of the dose of the BCTs, that is, when it was delivered, the frequency, and its intensity. Similarly, few studies reported unequivocally the number of contacts and/or the duration/length of each type of contact (email, text-message, phone call, etc). The only paper that described the intervention components with some detail and in a structured form (including almost all categories of the ontologies used in this review) was the NuLevel protocol.^45^

Few studies reported on tailoring features, and from the studies using this feature, only a few explained the decision process of personalized feedback and prompts (e.g. ^35^), going beyond the indication of the use of ‘personalized’ or ‘tailored’ or ‘individualized reports/feedback’. Only ‘NuLevel’, ‘NoHoW’ and ‘Motimate’ interventions broadly addressed the mechanics underlying the decisions of personalized feedback, there is a need for future studies to distinguish between automated feedback and human feedback (even if it is not face-to-face) and provide information about tailoring variables and the methods used for tailoring or personalization (e.g. data-driven).

Finally, most studies compared digitally delivered interventions (either as a stand-alone MoD or as a complementary platform) against other types of traditional MoDs (group, phone call, consultation plus digital). The testing of the differential effects of various digital MoDs and the other components was not addressed by any study. On the other hand, while most studies clearly reported BCTs and in some cases used standardized guidelines, there were serious limitations in the reporting of other interventions components, namely the ways through which the intervention content was delivered (i.e. MoDs) and its dose. In fact, very few studies systematically described the links between BCTs and the other intervention components. Intervention content underreporting undermines comparability across interventions and replication. Indeed, several researchers identified this gap before.^53–55^ This was the first review with the specific intent to provide an overarching mapping of how these components are brought together in DBCIs for WLM targeting PA, to inform us about the current state of evidence beyond the effectiveness.

### Strengths and limitations

This scoping review main strength is the classification of DBCIs components in a systematic manner using recommended taxonomies and ontologies, therefore contributing to the identification of knowledge gaps, and improvement of intervention reporting and design. This work allowed us to pinpoint two main gaps in the DBCI literature. First, the difficulty in extracting information from published literature in a systematic and objective manner, given the heterogeneity with which intervention components are described. Second, even when the components of the DBCI were reported, the links between these components were not fully described. Whether the intervention is effective or not, with no information on these links between the components we cannot know with a sufficient degree of certainty why the intervention outputs are as they are. If the ultimate goal of DBCIs/Digital behavioural research is to understand *what works, for whom, when, and why*, we need, as researchers, to standardize methods of reporting interventions’ components. Furthermore, the identification of the core components of DBCIs through the publication of the design protocols may contribute to the interaction between academia and industry. The ongoing efforts in behavioural science to develop standardized tools such as ontologies for developing and reporting digital interventions content will have a decisive contribution to identify which components of interventions influence the outcomes of interventions, therefore contributing to evidence synthesis and selection of core components in a parsimonious way.

Because ontologies for Dose and for Tailoring are not yet available, we used existing frameworks to guide our analysis.^24^ Tailoring and related approaches (e.g. personalization) have been conceptualized and described in a vast body of literature in the health domain (e.g. in health communication^56,57^). Nonetheless, there is no standardized and agreed classification of the features associated with tailoring, personalization and related approaches. This effort is currently being pursued by Human Behaviour Change Project working group.^23,58^ This endeavour will allow for an update of our work in the future.

We conducted a literature search in different bibliographic databases as recommended. This search would have been more comprehensive if Medical Subject Headings (MeSH) terms had been used for the PubMed database. We decided not to use these terms due to the latency with which articles are indexed to the MeSH. Further, we only included published RCTs studies. Expanding this search to computer science databases (e.g. ACM Digital library), app libraries and grey literature, could provide valuable information on how DBCIs are described in a different scientific field and to more detailed descriptions of interventions that are sometimes provided in unpublished documentation. Also, the specific focus of this review on RCTs may have contributed to the fact that only one fully digital and automated DBCI was found. It is possible that a search including other type of research designs, and real-world interventions and commercial programs would have found more fully automated studies. Future studies could investigate the effects of these types of DBCIs in weight management.

Another limitation we would like to highlight is that it was out of the scope of this review to examine which components and/or links between components were associated with the effects of interventions. Nonetheless, the extensive data extraction conducted allowed us to verify that the limited number of interventions and the limitation on the descriptions of its components would not allow us to conduct this analysis. Furthermore, we did not assess the methodological quality of the studies due to the nature of this review, focusing on the quality and congruence of the intervention content and components rather than the quality of the methodological procedures. Finally, in this review we coded information on behaviour change intervention components only from published study protocols and trial results because we were interested in critically appraising what is reported in published literature. This may be considered a limitation since, as found in recent research,^53^ detailed information about intervention characteristics is sometimes only provided in unpublished sources, for example, trial manuals. To support replication and evidence accumulation, we appeal to researchers to make their protocols as detailed as possible when it comes to the components of their interventions and provide public access to the content of their interventions (e.g. DBCI modules) through open access platforms such as Open Science Framework.^59^

## Conclusions

This scoping review identified which core components of DBCIs for WLM focusing on PA are reported, which links between components and BCTs are reported, and the level of detail of these reporting procedures. This is one of the first efforts to systematically describe a vast range of components of these DBCIs and identify in which domains the intervention development and reporting procedures need more attention. This review is also broad in scope as it identifies a range of modes of delivery beyond the typical digital platforms, including mobile interactions such as applications, phone calls or SMS. By identifying the extent by which components are adequately described, and the heterogeneity of this reporting procedures across studies, this work contributes to expand our knowledge on what is being done regarding DBCIs for WLM and what needs to be improved in future studies.

## Supplemental Material

sj-docx-1-dhj-10.1177_20552076221129089 - Supplemental material for What goes on in digital behaviour change interventions for weight loss maintenance targeting physical activity: A scoping review Click here for additional data file. Supplemental material, sj-docx-1-dhj-10.1177_20552076221129089 for What goes on in digital behaviour change interventions for weight loss maintenance targeting physical activity: A scoping review by Jorge Encantado, António L Palmeira, Carolina Silva, Falko F Sniehotta, R James Stubbs, Maria João Gouveia, Pedro J Teixeira, Berit L Heitmann and Marta M Marques in Digital Health

sj-docx-2-dhj-10.1177_20552076221129089 - Supplemental material for What goes on in digital behaviour change interventions for weight loss maintenance targeting physical activity: A scoping review Click here for additional data file. Supplemental material, sj-docx-2-dhj-10.1177_20552076221129089 for What goes on in digital behaviour change interventions for weight loss maintenance targeting physical activity: A scoping review by Jorge Encantado, António L Palmeira, Carolina Silva, Falko F Sniehotta, R James Stubbs, Maria João Gouveia, Pedro J Teixeira, Berit L Heitmann and Marta M Marques in Digital Health

sj-xlsx-3-dhj-10.1177_20552076221129089 - Supplemental material for What goes on in digital behaviour change interventions for weight loss maintenance targeting physical activity: A scoping review Click here for additional data file. Supplemental material, sj-xlsx-3-dhj-10.1177_20552076221129089 for What goes on in digital behaviour change interventions for weight loss maintenance targeting physical activity: A scoping review by Jorge Encantado, António L Palmeira, Carolina Silva, Falko F Sniehotta, R James Stubbs, Maria João Gouveia, Pedro J Teixeira, Berit L Heitmann and Marta M Marques in Digital Health

sj-xlsx-4-dhj-10.1177_20552076221129089 - Supplemental material for What goes on in digital behaviour change interventions for weight loss maintenance targeting physical activity: A scoping review Click here for additional data file. Supplemental material, sj-xlsx-4-dhj-10.1177_20552076221129089 for What goes on in digital behaviour change interventions for weight loss maintenance targeting physical activity: A scoping review by Jorge Encantado, António L Palmeira, Carolina Silva, Falko F Sniehotta, R James Stubbs, Maria João Gouveia, Pedro J Teixeira, Berit L Heitmann and Marta M Marques in Digital Health

sj-docx-5-dhj-10.1177_20552076221129089 - Supplemental material for What goes on in digital behaviour change interventions for weight loss maintenance targeting physical activity: A scoping review Click here for additional data file. Supplemental material, sj-docx-5-dhj-10.1177_20552076221129089 for What goes on in digital behaviour change interventions for weight loss maintenance targeting physical activity: A scoping review by Jorge Encantado, António L Palmeira, Carolina Silva, Falko F Sniehotta, R James Stubbs, Maria João Gouveia, Pedro J Teixeira, Berit L Heitmann and Marta M Marques in Digital Health

sj-xlsx-6-dhj-10.1177_20552076221129089 - Supplemental material for What goes on in digital behaviour change interventions for weight loss maintenance targeting physical activity: A scoping review Click here for additional data file. Supplemental material, sj-xlsx-6-dhj-10.1177_20552076221129089 for What goes on in digital behaviour change interventions for weight loss maintenance targeting physical activity: A scoping review by Jorge Encantado, António L Palmeira, Carolina Silva, Falko F Sniehotta, R James Stubbs, Maria João Gouveia, Pedro J Teixeira, Berit L Heitmann and Marta M Marques in Digital Health
