# Supplementary material for: What goes on in digital behaviour change interventions for weight loss maintenance targeting physical activity: A scoping review
Source: Digit Health. 2022 Nov 6;8:20552076221129089. doi: 10.1177/20552076221129089 (PMC9643762; doi:10.1177/20552076221129089)
Supplement: sj-docx-1-dhj-10.1177_20552076221129089 - Supplemental material for What goes on in digital behaviour change interventions for weight loss maintenance targeting physical activity: A scoping review [file sj-docx-1-dhj-10.1177_20552076221129089.docx]

**Search Terms**

technolog* OR digital OR web-based OR internet OR online OR ehealth OR mhealth OR app* OR mobile OR computer* OR smartphone) AND (‘weight loss maintenance’ OR ‘weight regain prevention’) AND (‘physical activity’ OR ‘sedentary behav*’ OR ‘exercise’).

**List of screened studies**

| 1. full text not available/Conference paper = 10 |
| --- |
| 2. not a weight loss maintenance intervention = 11 |
| 3. participants not required to have lost weight before intervention = 10 |
| 4. intervention didn't involve PA/exercise = 2 |
| 5. not digital/web-based intervention = 6  Table 1. List of screened studies |

| **Year** | **Author** | **Title** | **Decision** |
| --- | --- | --- | --- |
| 2018 | Alcantara-Aragon | Web Support for Weight-Loss Interventions: PREDIRCAM2 Clinical Trial Baseline Characteristics and Preliminary Results | Excluded - 3 |
| 2017 | Arnold | Provider interest in lifestyle tracking within the EHR: Data from the maintain-pc study | Excluded - 1 |
| 2016 | Arnold | Provider feedback on weight loss maintenance resources and a novel intervention using the electronic medical record: Results from the maintain-PC study | Excluded - 1 |
| 2018 | Arnold | Online lifestyle tracking only improves weight outcomes in conjunction with coaching support: Results from the maintain-PC study | Excluded - 1 |
| 2008 | Brantley | Design considerations and rationale of a multi-center trial to sustain weight loss: the weight loss maintenance trial | Included |
| 2016 | Brindal | Combining Persuasive Technology With Behavioral Theory to Support Weight Maintenance Through a Mobile Phone App: Protocol for the MotiMate App | Included (protocol: Brindal et al. 2019 - A Mobile Phone App Designed to Support Weight Loss Maintenance and Well-Being (MotiMate) - Randomized Controlled Trial) |
| 2019 | Brindal | A Mobile Phone App Designed to Support Weight Loss Maintenance and Well-Being (MotiMate): Randomized Controlled Trial | Included (see protocol study: Brindal et al. 2016 - Combining Persuasive Technology With Behavioral Th ... aintenance Through a Mobile Phone App - Protocol for the MotiMate App) |
| 2013 | Carnie | Randomized trial of nutrition education added to internet-based information and exercise at the work place for weight loss in a racially diverse population of overweight women | Excluded - 4 |
| 2011 | Castelnuovo | TECNOB study: Ad interim results of a randomized controlled trial of a multidisciplinary telecare intervention for obese patients with type-2 diabetes | Excluded - 2 |
| 2010 | Collins | Evaluation of a commercial web-based weight loss and weight loss maintenance program in overweight and obese adults: a randomized controlled trial | Included |
| 2013 | Coughlin | Behavioral Mediators of Treatment Effects in the Weight Loss Maintenance Trial | Included |
| 2016 | Eng | PCP weight loss assistance in a primary care population: Lessons learned from participants with recent intentional weight loss in the maintain-PC study | Excluded - 1 |
| 2019 | Espel-Huynh | Rationale and design for a pragmatic effectiveness-implementation trial of online behavioral obesity treatment in primary care | Included |
| 2015 | Evans | The NULevel trial of a scalable, technology-assisted weight loss maintenance intervention for obese adults after clinically significant weight loss: study protocol for a randomised controlled trial | Included |
| 2010 | Funk | Associations of Internet Website Use With Weight Change in a Long-term Weight Loss Maintenance Program | Included |
| 2013 | Gerber | Video telehealth for weight maintenance of African-American women | Included |
| 2009 | Gerber | Mobile phone text messaging to promote healthy behaviors and weight loss maintenance: a feasibility study | Excluded - 3 |
| 2012 | Greaney | Use of Email and Telephone Prompts to Increase Self-Monitoring in a Web-Based Intervention: Randomized Controlled Trial | Excluded - 2 |
| 2016 | Karppinen | Persuasive user experiences of a health Behavior Change Support System: A 12-month study for prevention of metabolic syndrome | Excluded - 3 |
| 2017 | Kwasnicka | N-of-1 study of weight loss maintenance assessing predictors of physical activity, adherence to weight loss plan and weight change | Excluded - 2 |
| 2012 | Lambourne | Weight management by phone conference call: A comparison with a traditional face-to-face clinic. Rationale and design for a randomized equivalence trial | Excluded - 5 |
| 2019 | Larsen | Perceived support during and after aweight loss maintenance intervention: Secondary analysis from the maintain-pc trial | Excluded - 1 |
| 2016 | Leahey | A randomized controlled trial testing an Internet delivered cost-benefit approach to weight loss maintenance | Included |
| 2010 | Martin | Efficacy of an e-Health intervention at promoting weight loss through remote delivery of services: Preliminary results from a randomized controlled trial | Excluded - 1 |
| 2010 | Maruyama | Effect of a worksite-based intervention program on metabolic parameters in middle-aged male white-collar workers: A randomized controlled trial | Excluded - 3 |
| 2013 | Mehring | Effects of a general practice guided web-based weight reduction program–results of a cluster-randomized controlled trial | Excluded - 3 |
| 2013 | Murphy | Weight-loss study in African-American Women: lessons learned from project take HEED and future, technologically enhanced directions | Excluded - 3 |
| 2015 | Nakata | Web-based intervention to promote weight loss maintenance using an activity monitor: Study design and 3-month interim report of a randomised controlled trial | Excluded - 1 |
| 2019 | Nakata | Web-based intervention to promote weight-loss maintenance using an activity monitor: A randomized controlled trial | Included |
| 2017 | Podina | An evidence-based gamified mHealth intervention for overweight young adults with maladaptive eating habits: study protocol for a randomized controlled trial | Excluded - 3 |
| 2019 | Ross | Week-to-week predictors of weight loss and regain | Excluded - 2 |
| 2017 | Scott | The development of the NoHoW trial for weight loss maintenance: Design, analyses, challenges and solutions | Excluded - 1 |
| 2019 | Scott | The NoHoW protocol: a multicentre 2x2 factorial randomised controlled trial investigating an evidence-based digital toolkit for weight loss maintenance in European adults | Included |
| 2013 | Shaw | Development of a Theoretically Driven mHealth Text Messaging Application for Sustaining Recent Weight Loss | Excluded - 5 |
| 2017 | Sniehotta | Effectiveness of a digitally delivered behavioural intervention for weight loss maintenance in obese adults: The randomised controlled NULevel trial | Excluded - 1 |
| 2019 | Sniehotta | Behavioural intervention for weight loss maintenance versus standard weight advice in adults with obesity: A randomised controlled trial in the UK (NULevel Trial) | Included |
| 2008 | Stevens | Design and implementation of an interactive website to support long-term maintenance of weight loss | Included |
| 2015 | Taylor | Determining how best to support overweight adults to adhere to lifestyle change: protocol for the SWIFT study | Excluded - 3 |
| 2011 | Thomas | E-mail contact as an effective strategy in the maintenance of weight loss in adults | Included |
| 2015 | Wang | Wearable Sensor/Device (Fitbit One) and SMS Text-Messaging Prompts to Increase Physical Activity in Overweight and Obese Adults: A Randomized Controlled Trial | Excluded - 3 |
| 2008 | Wing | Maintaining large weight losses: The role of behavioral and psychological factors | Included |
| 2018 | Yancy | Financial incentive strategies for maintenance of weight loss: results from an internet-based randomized controlled trial | Excluded - 4 |
| 2017 | Collins | Efficacy of Web-Based Weight Loss Maintenance Programs: A Randomized Controlled Trial Comparing Standard Features Versus the Addition of Enhanced Personalized Feedback over 12 Months | Included (hand searched) |
| **List from the search update from November 2019 to January 18, 2021** | | | |
| 2020 | Leahey | Episodic Future Thinking, Delay Discounting, and Exercise During WeightLoss Maintenance: The PACE Trial | Excluded – 5 (Maintenance phase do not use of digital/web-based intervention) |
| 2019 (Nov) | Yancy | Effect of Escalating Financial Incentive Rewards on Maintenance of Weight Loss: A Randomized Clinical Trial | Excluded - 5 |
| 2020 | Knäuper | The effects of if-then plans on weight loss: results of the 24-month follow-up of the McGill CHIP Healthy Weight Program randomized controlled trial | Excluded - 2 |
| 2020 | Voils | Protocol for Partner2Lose: A randomized controlled trial to evaluate partner involvement on long-term weight loss | Excluded - 2 |
| 2020 | Kwasnicka | Theory-based digital intervention to promote weight loss and weight loss maintenance (Choosing Health): protocol for a randomised controlled trial | Excluded – 3 (Main intervention do not include weight losers) |
| 2020 | Dodge | Physical activity goals among patients in a primary care setting | Excluded - 2 |
| 2020 | Hernandez Reyes | Push Notifications From a Mobile App to Improve the Body Composition of Overweight or Obese Women: Randomized Controlled Trial | Excluded - 2 |
| 2020 | Ho | Community-based weight loss programme targeting overweight Chinese adults with pre-diabetes: study protocol of a randomised controlled trial | Excluded - 2 |
| 2019 (Novr) | Holmes | WeightMentor, bespoke chatbot for weight loss maintenance: Needs assessment & Development | Excluded - 1 |
| 2020 | Mailey | Promoting Strength Training Among Baby Boomers: Message Framing Effects on Motivation and Behavior | Excluded - 2 |
| 2020 | Stegar | Intermittent and continuous energy restriction result in similar weight loss, weight loss maintenance, and body composition changes in a 6 month randomized pilot study | Excluded - 2 |
| 2019 (Nov) | Smith | The community-based prevention of diabetes (ComPoD) study: a randomised, waiting list controlled trial of a voluntary sector-led diabetes prevention programme | Excluded - 5 |
| 2020 | Taheri | Effect of intensive lifestyle intervention on bodyweight and glycaemia in early type 2 diabetes (DIADEM-I): an open-label, parallel-group, randomised controlled trial | Excluded - 5 |

Table 2. Interrater percent agreement on Dose of Intervention

|  | Percent Agreement | N Agreements | N Disagreements | N Cases | N Decisions |
| --- | --- | --- | --- | --- | --- |
| Design | 100 | 11 | 0 | 11 | 22 |
| Amount | 100 | 11 | 0 | 11 | 22 |
| Number of Contacts | 45.45455 | 5 | 6 | 11 | 22 |
| Amount of Materials | 81.81818 | 9 | 2 | 11 | 22 |
| BCTs | 90.90909 | 10 | 1 | 11 | 22 |
| Frequency of Contacts | 81.81818 | 9 | 2 | 11 | 22 |
| Frequency of delivery of meterials/modules | 90.90909 | 10 | 1 | 11 | 22 |
| Frequency of delivery of BCTs | 90.90909 | 10 | 1 | 11 | 22 |
| Overall duration of contacts | 63.63636 | 7 | 4 | 11 | 22 |
| Duration of each contact | 63.63636 | 7 | 4 | 11 | 22 |
| Duration of delivery of each bct | 90.90909 | 10 | 1 | 11 | 22 |
| Order of components | 100 | 11 | 0 | 11 | 22 |
| Regularity | 36.36364 | 4 | 7 | 11 | 22 |

Table 3. Interrater percent agreement on Mode of Delivery of Intervention

|  | Percent Agreement | N Agreements | N Disagreements | N Cases | N Decisions |
| --- | --- | --- | --- | --- | --- |
| Face | 90.90909 | 10 | 1 | 11 | 22 |
| Distant | 100 | 11 | 0 | 11 | 22 |
| Printed Material | 100 | 11 | 0 | 11 | 22 |
| Technology Delivered | 100 | 11 | 0 | 11 | 22 |
| Digital Content Type | 90.90909 | 10 | 1 | 11 | 22 |
| Environment Change Object | 90.90909 | 10 | 1 | 11 | 22 |
| Type of Interaction | 100 | 11 | 0 | 11 | 22 |
| Direction of Interaction | 63.63636 | 7 | 4 | 11 | 22 |
| Dynamics of Interaction | 72.72727 | 8 | 3 | 11 | 22 |
| Feature of Interaction | 90.90909 | 10 | 1 | 11 | 22 |
| Format of Interaction | 100 | 11 | 0 | 11 | 22 |
| Gamification | 90.90909 | 10 | 1 | 11 | 22 |

Table 3. Interrater percent agreement on Behaviour Change Techniques

|  | Percent Agreement | N Agreements | N Disagreements | N Cases | N Decisions |
| --- | --- | --- | --- | --- | --- |
| 1.1. Goal setting (behavior) | 90.90909 | 10 | 1 | 11 | 22 |
| 1.2. Problem solving | 100 | 11 | 0 | 11 | 22 |
| 1.3. Goal setting (outcome) | 90.90909 | 10 | 1 | 11 | 22 |
| 1.4. Action planning | 100 | 11 | 0 | 11 | 22 |
| 1.5. Review behavior goal(s) | 100 | 11 | 0 | 11 | 22 |
| 1.6. Discrepancy between current behavior and goal | 100 | 11 | 0 | 11 | 22 |
| 1.7. Review outcome goal(s) | 100 | 11 | 0 | 11 | 22 |
| 1.8. Behavioral contract | 100 | 11 | 0 | 11 | 22 |
| 1.9. Commitment | 100 | 11 | 0 | 11 | 22 |
| 2.1. Monitoring of behavior by others without feedback | 100 | 11 | 0 | 11 | 22 |
| 2.2. Feedback on behavior | 81.81818 | 9 | 2 | 11 | 22 |
| 2.3. Self-monitoring of behavior | 100 | 11 | 0 | 11 | 22 |
| 2.4. Self-monitoring of outcome(s) of behavior | 100 | 11 | 0 | 11 | 22 |
| 2.5. Monitoring of outcome(s) of behavior without feedback | 100 | 11 | 0 | 11 | 22 |
| 2.6. Biofeedback | 100 | 11 | 0 | 11 | 22 |
| 2.7. Feedback on outcome(s) of behavior | 90.90909 | 10 | 1 | 11 | 22 |
| 3.1. Social support (unspecified) | 81.81818 | 9 | 2 | 11 | 22 |
| 3.2. Social support (practical) | 90.90909 | 10 | 1 | 11 | 22 |
| 3.3. Social support (emotional) | 100 | 11 | 0 | 11 | 22 |
| 4.1. Instruction on how to perform the behavior | 90.90909 | 10 | 1 | 11 | 22 |
| 4.2. Information about antecendents | 100 | 11 | 0 | 11 | 22 |
| 4.3. Re-attribution | 100 | 11 | 0 | 11 | 22 |
| 4.4. Behavioral experiments | 100 | 11 | 0 | 11 | 22 |
| 5.1. Information about health consequences | 90.90909 | 10 | 1 | 11 | 22 |
| 5.2. Salience of consequences | 100 | 11 | 0 | 11 | 22 |
| 5.3. Information about social and environmental consequences | 100 | 11 | 0 | 11 | 22 |
| 5.4. Monitoring of emotional consequences | 100 | 11 | 0 | 11 | 22 |
| 5.5. Anticipated regret | 100 | 11 | 0 | 11 | 22 |
| 5.6. Information about emotional consequences | 100 | 11 | 0 | 11 | 22 |
| 6.1. Demonstration of the behavior | 100 | 11 | 0 | 11 | 22 |
| 6.2. Social comparison | 100 | 11 | 0 | 11 | 22 |
| 6.3. Information about others’ approval | 100 | 11 | 0 | 11 | 22 |
| 7.1. Prompts/cues | 90.90909 | 10 | 1 | 11 | 22 |
| 7.2. Cue signalling reward | 100 | 11 | 0 | 11 | 22 |
| 7.3. Reduce prompts/cues | 100 | 11 | 0 | 11 | 22 |
| 7.4. Remove access to the reward | 100 | 11 | 0 | 11 | 22 |
| 7.5. Remove aversive stimulus | 100 | 11 | 0 | 11 | 22 |
| 7.6. Satiation | 100 | 11 | 0 | 11 | 22 |
| 7.7. Exposure | 100 | 11 | 0 | 11 | 22 |
| 7.8. Associative learning | 100 | 11 | 0 | 11 | 22 |
| 8.1. Behavioral practice/rehearsal | 100 | 11 | 0 | 11 | 22 |
| 8.2. Behavior substitution | 100 | 11 | 0 | 11 | 22 |
| 8.3. Habit formation | 100 | 11 | 0 | 11 | 22 |
| 8.4. Habit reversal | 100 | 11 | 0 | 11 | 22 |
| 8.5. Overcorrection | 100 | 11 | 0 | 11 | 22 |
| 8.6. Generalisation of target behavior | 100 | 11 | 0 | 11 | 22 |
| 8.7. Graded tasks | 100 | 11 | 0 | 11 | 22 |
| 9.1. Credible source | 100 | 11 | 0 | 11 | 22 |
| 9.2. Pros and cons | 100 | 11 | 0 | 11 | 22 |
| 9.3. Comparative imagining of future outcomes | 100 | 11 | 0 | 11 | 22 |
| 10.1. Material incentive (behavior) | 100 | 11 | 0 | 11 | 22 |
| 10.2. Material reward (behavior) | 100 | 11 | 0 | 11 | 22 |
| 10.3. Non-specific reward | 100 | 11 | 0 | 11 | 22 |
| 10.4. Social reward | 100 | 11 | 0 | 11 | 22 |
| 10.5. Social incentive | 100 | 11 | 0 | 11 | 22 |
| 10.6. Non-specific incentive | 100 | 11 | 0 | 11 | 22 |
| 10.7. Self-incentive | 100 | 11 | 0 | 11 | 22 |
| 10.8. Incentive (outcome) | 100 | 11 | 0 | 11 | 22 |
| 10.9. Self-reward | 90.90909 | 10 | 1 | 11 | 22 |
| 10.10. Reward (outcome) | 100 | 11 | 0 | 11 | 22 |
| 10.11. Future punishment | 100 | 11 | 0 | 11 | 22 |
| 11.1. Pharmacological support | 100 | 11 | 0 | 11 | 22 |
| 11.2. Reduce negative emotions | 100 | 11 | 0 | 11 | 22 |
| 11.3. Conserving mental resources | 100 | 11 | 0 | 11 | 22 |
| 11.4. Paradoxical instructions | 100 | 11 | 0 | 11 | 22 |
| 12.1. Restructuring the physical environment | 100 | 11 | 0 | 11 | 22 |
| 12.2. Restructuring the social environment | 100 | 11 | 0 | 11 | 22 |
| 12.3. Avoidance/reducing exposure to cues for the behavior | 100 | 11 | 0 | 11 | 22 |
| 12.4. Distraction | 100 | 11 | 0 | 11 | 22 |
| 12.5. Adding objects to the environment | 81.81818 | 9 | 2 | 11 | 22 |
| 12.6. Body changes | 100 | 11 | 0 | 11 | 22 |
| 13.1. Identification of self as role model | 100 | 11 | 0 | 11 | 22 |
| 13.2. Framing/reframing | 100 | 11 | 0 | 11 | 22 |
| 13.3. Incompatible beliefs | 100 | 11 | 0 | 11 | 22 |
| 13.4. Valued self-identify | 100 | 11 | 0 | 11 | 22 |
| 13.5. Identity associated with changed behavior | 100 | 11 | 0 | 11 | 22 |
| 14.1. Behavior cost | 100 | 11 | 0 | 11 | 22 |
| 14.2. Punishment | 100 | 11 | 0 | 11 | 22 |
| 14.3. Remove reward | 100 | 11 | 0 | 11 | 22 |
| 14.4. Reward approximation | 100 | 11 | 0 | 11 | 22 |
| 14.5. Rewarding completion | 100 | 11 | 0 | 11 | 22 |
| 14.6. Situation-specific reward | 100 | 11 | 0 | 11 | 22 |
| 14.7. Reward incompatible behavior | 100 | 11 | 0 | 11 | 22 |
| 14.8. Reward alternative behavior | 100 | 11 | 0 | 11 | 22 |
| 14.9. Reduce reward frequency | 100 | 11 | 0 | 11 | 22 |
| 14.10. Remove punishment | 100 | 11 | 0 | 11 | 22 |
| 15.1. Verbal persuasion about capability | 100 | 11 | 0 | 11 | 22 |
| 15.2. Mental rehearsal of successful performance | 100 | 11 | 0 | 11 | 22 |
| 15.3. Focus on past success | 100 | 11 | 0 | 11 | 22 |
| 15.4. Self-talk | 100 | 11 | 0 | 11 | 22 |
| 16.1. Imaginary punishment | 100 | 11 | 0 | 11 | 22 |
| 16.2. Imaginary reward | 100 | 11 | 0 | 11 | 22 |
| 16.3. Vicarious consequences | 100 | 11 | 0 | 11 | 22 |

Table 4. Interrater percent agreement on Mechanisms of Action

|  | Percent Agreement | N Agreements | N Disagreements | N Cases | N Decisions |
| --- | --- | --- | --- | --- | --- |
| Attitude towards the behavior | 100 | 11 | 0 | 11 | 22 |
| Behavioral cueing | 100 | 11 | 0 | 11 | 22 |
| Behavioral regulation | 90.90909 | 10 | 1 | 11 | 22 |
| Beliefs about capabilities | 81.81818 | 9 | 2 | 11 | 22 |
| Beliefs about consequences | 100 | 11 | 0 | 11 | 22 |
| Environmental context and resources | 90.90909 | 10 | 1 | 11 | 22 |
| Emotion | 90.90909 | 10 | 1 | 11 | 22 |
| Feedback processes | 100 | 11 | 0 | 11 | 22 |
| General attitudes/beliefs | 100 | 11 | 0 | 11 | 22 |
| Goals | 100 | 11 | 0 | 11 | 22 |
| Intention | 100 | 11 | 0 | 11 | 22 |
| Knowledge | 100 | 11 | 0 | 11 | 22 |
| Memory, attention, and decision Processes | 90.90909 | 10 | 1 | 11 | 22 |
| Motivation | 90.90909 | 10 | 1 | 11 | 22 |
| Perceived susceptibility/ vulnerability | 100 | 11 | 0 | 11 | 22 |
| Needs | 100 | 11 | 0 | 11 | 22 |
| Reinforcement | 81.81818 | 9 | 2 | 11 | 22 |
| Self-image | 100 | 11 | 0 | 11 | 22 |
| Skills | 81.81818 | 9 | 2 | 11 | 22 |
| Social influences | 90.90909 | 10 | 1 | 11 | 22 |
| Subjective norms | 100 | 11 | 0 | 11 | 22 |
| Social learning/imitation | 100 | 11 | 0 | 11 | 22 |
| Social/professional role and identity | 100 | 11 | 0 | 11 | 22 |
| Values | 100 | 11 | 0 | 11 | 22 |

Table 5. Interrater percent agreement on Tailoring

|  | Percent Agreement | N Agreements | N Disagreements | N Cases | N Decisions |
| --- | --- | --- | --- | --- | --- |
| Tailoring | 81.81818 | 9 | 2 | 11 | 22 |
