# Supplementary material for: What goes on in digital behaviour change interventions for weight loss maintenance targeting physical activity: A scoping review
Source: Digit Health. 2022 Nov 6;8:20552076221129089. doi: 10.1177/20552076221129089 (PMC9643762; doi:10.1177/20552076221129089)
Supplement: sj-docx-2-dhj-10.1177_20552076221129089 - Supplemental material for What goes on in digital behaviour change interventions for weight loss maintenance targeting physical activity: A scoping review [file sj-docx-2-dhj-10.1177_20552076221129089.docx]

Supplementary File 2. Summary of interventions’ characteristics

| **Article/Intervention** | **Type Contact** | **Behaviour Change Techniques** | **Mechanisms of Action** | **Mode of Delivery** | | | **Dose** | **Tailoring** |
| --- | --- | --- | --- | --- | --- | --- | --- | --- |
|  |  |  |  | **Cross Cutting Entities** | **Format** | **Modules** |  |  |
| -Brindal 2019 (RCT)  -Brindal 2016 (protocol)  (Motimate)  Diet and PA | -distant  -mobile first (app)  -text messages  -audio (coping call)  -automate email | -2.2 Feedback on behaviour  -2.3 Self-monitoring of behaviour  -2.4 Monitoring of Outcome of Behaviour  -2.7 Feedback on outcome of behaviour  -3.1 social support (unspecified)  -5.4 Monitoring of emotional consequences  -7.1 Prompts/cues [Behavioural prompts]  -11.2 Reduce negative emotions  -12.3 Avoidance/reducing exposure to cues for the behaviour  -12.4 distraction  -13.2 Framing/Reframing  -13.4 Valued self-identify  -15.3 Focus on past success | -behavioural regulation  -emotion  -perceived susceptibility/vulnerability  -goals  -feedback processes  -optimism  -skills  -motivation  -needs  -optimism  -beliefs about capabilities  -self-image | -individual  -one way  -reciprocal  -synchronous  -push  -pull | -audio  -text  -infographic | *unclear  -emotion regulation digital workshop | -permanent  -weekly feedback  -5 clinic visits (assessments)  -low intensity but frequent interactions (<10 seconds)  -daily push notifications to enter data | -feedback is based on existing definitions of WLM  -if weight is gained, “just in time” phone call with professional  -coping strategies in predefined order using an algorithm assessing user appraisals of the hassle |
| -Collins et al., 2010  -Collins et al., 2019  (CTRN12610000197033)  Diet and PA | -distant  -desktop  -mobile (sms/calls)  -pedometers  -audio  -sms  -email  -website | -1.1. Goal setting behaviour  -1.3. Goal setting outcome  -2.2 Feedback on behaviour  -2.3. Self-monitoring of behaviour  -2.4. Self-monitoring of outcomes of behaviour  -2.7. Feedback on outcomes of behaviour  -3.1. Social support unspecified  -3.2 Social support (practical)  -7.1. Prompts/cues  -10.10. Reward outcome | -behavioural regulation  -environmental context and resources  -social influences  -goals  -skills  -beliefs about consequences  -knowledge  -social learning/imitation  -social influences | -individual  -one-way  -reciprocal  -synchronous  -asynchronous  -push | -audio  -text  -infographic | To target mediators:  -self-efficacy  -outcome expectations  -modelling  -social support | -weekly feedback  -monthly feedbacks  -weekly prompts for self-monitoring | -personalized reports  -questionnaires to help identify and prioritize behaviours  -feedback on weigh-ins “warnings” |
| -Coughlin, 2013  -Brantley, 2008  -Funk, 2010  -Steven, 2008  (WLM Website BFS)  Diet and PA | -face-to-face  -distant  -email  -website  -audio (interactive phone call) | -1.1 Goal setting behaviour  -1.2 Problem Solving  -1.4 Action planning  -2.2 Feedback on behaviour  -2.3 Self monitoring behaviour  -2.4 feedback on outcome  -3.1 Social support  -5.1 Information about health consequences  -7.1 Prompts/cues  -9.1 Credible source | -skills  -behavioural regulation  -reinforcement  -beliefs about capabilities  -environmental context and resources  -social influences | -synchronous  -asynchronous  -reciprocal  -one way  -push  -pull  -group (orientation session) | -audio  -text  -infographic | -self-monitoring  -feedback  -problem-solving  -social support  -relapse prevention | -unlimited/continuous  -encouraged weekly contacts | -personally tailored goal setting and problem-solving programs  -automated feedback in the form of weight and exercise graphs, personalized messages, and tailored progress reports |
| -Espel-Huynn, 2019  (Refresher Weight Loss)  Diet and PA | -distant  -desktop  -website | -1.1. Goal setting behaviour  -1.2. Problem solving  -1.3. Goal setting outcome  -1.9 Commitment  -2.3. Self-monitoring of behaviour  -2.4. Self-monitoring of outcomes of behaviour  -2.7. Feedback on outcomes of behaviour  -5.1 Information about health consequences  -8.7 Graded tasks | -no MoAs reported | -individual  -one-way  -asynchronous  -pull | -text  -video  -infographic | -intro to maintain.  -commit. to WM and personal values  -understand metabolism and increase lifestyle PA  -eating a healthy breakfast  -changing “Yes, but” to “Yes, and”  -eating a healthy dinner  -manag. cravings and urges  -eating mindfully  -assertive commun. for WM | -9= 1 in the 1st month, weekly on month 4 and 7  -Week automated feedback after course/lesson | -no tailoring reported |
| -Evans 2015 (Protocol)  -Sniehotta 2019 (RCT)  (NuLevel)  Diet and PA | -distant  -face-to-face (1st session)  -printed material  -mobile (sms)  -desktop  -scale and activity monitor  -audio call (if requested)  -text messages  -web-based platform | -1.1 Goal setting (Behaviour)  -1.2 Problem solving  -1.3 Goal setting (outcome)  -1.4 Action planning  -1.5 Review of behavioural goals  -1.7 Review of outcome goal  -2.2 Feedback on behaviour  -2.3 Self-monitoring of behaviour  -2.4 Self-monitoring of behavioural outcome  -2.7 Feedback on outcome  -3.1 Social support (unspecified)  -3.2 Social support (practical)  -4.1 Instruction on how to perform the behaviour  -5.1 Information about health consequences  -7.1 Prompts/cues  -10.4 Social reward  -12.2 Plan social support/social change  -12.3 Avoidance/reducing exposure to cues for the behaviour  -12.5 Adding objects to the environment  -15.3 Prompting focus on past success | -knowledge  -goals  -self-efficacy  -behavioural regulation  -behavioural cueing  -memory, attention, decision process  -motivation  -self-Image  -values  -skills  -environmental context and resources  -social influences  -feedback processes  -needs  -beliefs about capabilities | -individual  -one way  -reciprocal  -synchronous  -asynchronous  -push  -pull | -text  -infographic | -6 face-to-face modules (1st session) | -1st face session- 60 to 90 mins  -305 sms  -daily feedback  -sms if no info reported | -tailoring of intervention components based on progress  -traffic light system to adjust intervention intensity  -automated and tailored feedback |
| -Gerber, 2013  (Exercise your Faith)  Diet and PA | -distant  -face-to-face (orientation)  -video  -audio  -email  -text  -television | -1.2 Problem solving  -3.1 Social Support  -4.1 Instruction on how to perform behaviour  -6.1 Demonstration of the behaviour  -7.1 Prompts/cues  -12.1 Restructuring the physical environment  -12.5 Adding objects to the environment | -no MoAs reported | -individual  -one way  -reciprocal (if asked)  -synchronous (if asked)  -asynchronous  -push  -pull | -audio  -video  -text | Video channels:  -informational  -beginner exercise videos  -advanced exercise videos  Calls:  -support problem solving  -prevent relapse  -sustained motivational efforts | -monthly telephone contact  -9 calls  -calls lasted 10-12 mins  -videos from 5 to 20 mins | -no tailoring reported |
| -Leahey, 2016  (Providence)  Diet and PA | -face-to-face  -distant  -desktop  -email | -1.1. Goal setting behaviour  -1.2. Problem solving  -1.3. Goal setting outcomes  -1.4. Action planning  -2.3. Self-monitoring of behaviour  -2.4. Self-monitoring of outcomes of behaviour  -4.1. Instructions on how to perform behaviour  -7.1. Prompts/cues  -10.1 Material incentive (behaviour)  -10.2. Material reward (behaviour)  -10.4 Social Reward  -10.10. Reward outcome  -14.6. Situation-specific reward | -beliefs about consequences  - reinforcement  -social influences  -environmental context and resources  -behavioural regulation | -individual  -dyadic (peer condition)  -group session (both)  -one way  -synchronous  -asynchronous  -push  -pull | -text  -gamification (irregular monetary incentives) | *unclear | -1 group session  -weekly emails (10 months)  -weekly monetary reinforcement upon data submission (weight and PA)  -Weekly reminders to submit SM information | -components designed to address the high cost–benefit ratio thought to undermine weight loss maintenance  -to decrease boredom or “costs” of long-term adherence, the maintenance program included a variety of different evidence-based strategies for weight loss maintenance  -Given that self-monitoring is consistently associated with better maintenance outcomes, both the social and financial reinforcers were provided contingent on self-monitoring. |
| -Nakata, 2019  (UMIN)  PA | -distant  -mobile  -desktop  -weight scale  -activity monitor  -audio call (reminder)  -email (reminder)  -website | -1.1. Goal setting behaviour  -2.2. Feedback on behaviour  -2.3. Self-monitoring of behaviour  -2.4. Self-monitoring of outcomes of behaviour  -2.7. Feedback on outcomes of behaviour  -3.1. Social support unspecified  -8.7 Graded tasks  -12.5 Adding objects to the environment | -no MoAs reported | -individual  -one way  -reciprocal  -synchronous  -asynchronous  -push  -pull | -audio  -text  -infographic | -unclear | -monthly personalized feedback (approximately 350–450 Japanese characters, corresponding to 200–250 words in English) for 24 months | -provided monthly personalized feedback  -goals were set individually with planned gradual increases |
| -Scott, 2019  (NoHoW Trial)  Diet and PA | -desktop  -device (scale and activity tracker)  -website | -2.3 Self-monitoring of behaviour  -2.4 Self-monitoring of outcome  -2.7 Feedback on outcome of behaviour  -7.1 Prompts/cues  -12.5 Adding objects to the environment | -behavioural regulation  -emotion  -motivation | -individual  -one way  -asynchronous  -push  -pull | -audio  -video  -text  -infographics  -gamification (quizzes, mini-apps) | Arm 2:  -8 modules  Arm 3:  -7 modules  Arm 4:  -15 modules | Arm 2:  -18+3 weekly emails  Arm 3:  -17+5 weekly emails  Arm 4:  -18+8 weekly emails | -individualized feedback component of arm 2 and arm 4 automatically provided by the toolkit in response to weight trajectory  -feedback is generated from data extracted  -feedback displayed in the toolkit as set of short statements |
| -Thomas, 2011  (Weight Loss Clinics)  Diet and PA | -distant  -desktop  -email | -2.4. Self-monitoring of outcomes of behaviour  -4.1. Instruction on how to perform the behaviour  -7.1. Prompts/cues | -no MoAs reported | -individual  -one way  -asynchronous | -text | -dietary  -behavioural  -exercise | -weekly emails for 6 months (26 emails) | -no tailoring reported |
| -Wing, 2008  (Stop Regain)  Diet and PA | -distant  -printed material (toolkit)  -desktop  -device scale  -email  -website  -pedometer (toolkit) | -1.2. Problem solving  -2.4. Self-monitoring of outcomes of behaviour  -3.1 Social support unspecified  -3.2 Social support practical  -4.1. Instructions on how to perform behaviour  -10.10. Reward outcome | -behavioural regulation | -individual  -one way  -asynchronous  -pull  -synchronous  -reciprocal (chat) | -text | -internet chat rooms focused on issues related to WLM and recommended strategies  -toolkit (red zone): self-monitoring diaries, personal WL success stories, info book, meal replacement products | -immediate positive messages  -gifts monthly  -weekly tips -reported their weekly weight and physical-activity data  -4 initial weekly meetings followed by monthly until the 18th month (group meetings - chat room) | -strategies delivered based on weight maintenance (green) or weight gain (yellow/red) |

Supplementary Figure 1. Presence of behaviour change techniques

Supplementary Figure 2. Presence of mechanisms of action

Supplementary Figure 3. Presence of modes of delivery

Supplementary Figure 4. Information regarding the dose of the intervention
