# Supplementary material for: What goes on in digital behaviour change interventions for weight loss maintenance targeting physical activity: A scoping review
Source: Digit Health. 2022 Nov 6;8:20552076221129089. doi: 10.1177/20552076221129089 (PMC9643762; doi:10.1177/20552076221129089)
Supplement: sj-docx-5-dhj-10.1177_20552076221129089 - Supplemental material for What goes on in digital behaviour change interventions for weight loss maintenance targeting physical activity: A scoping review [file sj-docx-5-dhj-10.1177_20552076221129089.docx]

Supplementary File 5. BCTs and MoAs extracted descriptions

**BCTs Descriptions**

**Brindal et al 2016 [Protocol]**

**2.2 Feedback on behaviour** (“Data from monitoring tools are collated into summary graphs to allow the users to reflect on their behaviors and identify potential patterns, as illustrated in Figure 1E. “ “Once a week a behavioral review is also released that summarizes the user’s weekly performance and gives feedback on which category a user is doing well on as well as their ”focus” area (i.e. an area where they are performing poorly)”)

**2.3 Self-monitoring of behaviour** (“Setting and reviewing goals are core features of most behavioral treatments which would not be possible without a level of behavioral monitoring. Monitoring tools were designed to encourage low intensity (<10seconds) and frequent interactions, as can be seen in Figure1A-D, are at the forefront of the user experience as ‘home page’ of the app.”); (“Figure 1Dshows how automatic textual feedback is given on exercise records based on a series of rules formed using the Australian National Physical Activity Guidelines for adults and how many entries the user had made for the day.”)

**2.4 Monitoring of Outcome of Behaviour [Weight monitoring feature]** (“Real-time two-way feedback is displayed below the weight data as it is entered.This feedback is tailored based on existing definitions of weight maintenance: maintaining, danger zone and gaining [26].Classification into one of these categories determines the nature of the textual feedback that people are given as well as the color of the weight display which, as can be seen in Figure 1A, progresses from green (maintaining) to dark green (danger zone) to grey (gaining).

**2.7 Feedback on outcome of behaviour** (“As participants enter the danger zone, the short automated message contains content designed to motivate them to stay on track. If they enter a gaining zone, the tone of these messages changes, remaining encouraging but becoming more directive, and an email is also sent to inform the administrator of the possible need for further intervention.Examples of messages for maintaining, danger zone and gaining messages respectively are: “Well done! You have maintained your starting weight. Keep it up,” “You are a little heavier than your starting weight. Just a bit more effort this week and you will be back on track,” and “You've gained some weight, try reviewing your lifestyle habits and keep keeping track of your weight.”)

**3.1 social support (unspecified)** (eg, “Talk with someone for advice”)

**5.4 Monitoring of emotional consequences** (Emotional monitoring “ track their moods, so that they can see any link between emotional states and other behaviors (in summary sections) but also to allow a point of EMI for the coping behavioral support tool (...) Mood states included were designed to capture pleasant and unpleasant moods as well as moods with high versus low arousal. A user also identifies emotional intensity from 1-10”); “Stress (captured independently tomood) is measured through an intensity bar.”

**7.1 Prompts/cues [Behavioural prompts]** (“MotiMate prompts participants through push notification messages to enter data daily and review feedback weekly.” [Monitoring and Reviewing])

**11.2 Reduce negative emotions** (“Psychological risk is calculated by summing the intensity scores for each moodentered each day (with pleasant and unpleasant moods associated with positive and negative scores respectively). A summed negative score is used to indicate an “unhappy” mood day. The system sends an alert for a severely unhappy day (a summed score 3 standard deviations from the mean) or prolonged“unhappy” days (7 consecutive days).” “A range of coping strategies are presented in a predefined order using an algorithm assessing user appraisals of the hassle, including the immediacy of the issue, a user’s perceived feeling of control and whether or not they want to fix the issue.) Strategies are grouped generally into categories)

**12.3 Avoidance/reducing exposure to cues for the behaviour** (“Escape/avoidant strategies could be associated with negative psychological symptoms [35] and therefore always appear at the bottom of this menu.”)

**12.4 distraction** (eg, “Avoid the issue”)

**13.2 Framing/Reframing** (Emotional or cognitive strategies (eg, “Change my thinking about it”) versus active strategies (eg, “Make a plan”).

**13.4 Valued self-identify** (personal strengths (eg, “Reflect on and use own strengths”)

**15.3 Focus on past success** (“Once a person has workshopped their coping strategies, Figure 1B shows how the data are summarized on their home screen once a person has workshopped their coping strategies. Terms such as strategies and ‘how I handled it’ are presented to users in an effort to avoid negative pre-conceptions related to ‘coping’”)

**Tailoring**

“motivating messages, personalized feedback in monitoring tools (as described above)” “Daily motivational messages were constructed based on inspirational quotes and thoughts to inspire optimism and the potential for higher resilience and problem-solving ability”

**Collins et al., 2010; 2017**

**Online program**

(“These participants had access to the online program described above plus received **automated, computer-generated, personalized feedback** based on their diet and physical activity diaries, their use or lack of use of the standard website features, and the level of success of their weight loss.”)

**1.1. Goal setting behaviour** (“Individualised daily Calorie targets to facilitate 0.5- 1 kg weight loss per week, based on participants weight, height and activity level”)

**1.3. Goal setting outcome [ MoD website - passive]** (“Goal setting options and graphical display of changes in body measurement data and body (BMI) silhouette.”)

**1.3. Goal setting outcome [ MoD website - active]** (“A personalised enrolment report (system-generated) which suggests weight loss goals and prioritizes key behaviours the user will need to change to be successful at weight maintenance”)

**2.2 Feedback on behaviour** (Weekly automated (system-generated) personalized feedback for key elements of nutrition and physical activity levels based on entries made in the online diary)

**2.3. Self-monitoring of behaviour** (“Online food and exercise diaries and search engines to facilitate entry of food data”)

**2.4. Self-monitoring of outcomes of behaviour** (“Self-monitoring of reported body weight, waist and hip girths, with automated weekly reminders for entering weight”)

**2.7. Feedback on outcomes of behaviour [MoD unclear - active]** (“Weekly (system-generated) personalized feedback reporting on the level of success of their weight loss journey, and general use of website features”)

**2.7. Feedback on outcomes of behaviour [ MoD website - passive]** (“Goal setting options and graphical display of changes in body measurement data and body (BMI) silhouette.”)

**3.1. Social support unspecified** (“Social support via online forums”)

**3.2 Social support (practical) [from staff)** (weekly educational tips and challenges)

**7.1. Prompts/cues [for self-monitoring of outcomes of behaviour]** (“Self-monitoring of reported body weight, waist and hip girths, with automated weekly reminders for entering weight”)

**7.1. Prompts/cues [for education]** (“weekly email newsletters notifying the user of new content relevant to their point in the program”; Reminder schedule for compliance to the diary, site visitations and a weekly weigh-in escalating with urgency (email, text messages and personal phone calls).)

**M1: Basic maintenance of lost weight program**

(Continued access to the online program for a further 12 months, including online forum access)

**1.3. Goal setting outcome** (“Adjusted daily Calorie target for weight maintenance.”)

**2.4. Self-monitoring of outcomes of behaviour & 7.1. Prompts/cues** (“Weekly email and phone text prompts to continue entering body weight and other body measurements.”)

**2.7. Feedback on outcome of behaviour** (“Maintenance-specific feedback on weigh-ins including warnings if the user’s weight is creeping up.”)

**3.1. Social support unspecified** (“Continued access to the online program for a further 12 months, including online forum access.”)

**10.10. Reward outcome** (“A certificate to celebrate the achievement of goal weight or reaching the maintenance phase.”)

**M2: Enhanced maintenance of lost weight program**

“All of the M1 program plus...”

**2.2. Feedback on behaviour 2.7. Feedback on outcomes of behaviour** (“Weekly and monthly system-generated, personalized reports summarizing energy balance and weight loss progress, website activity, and achievement of nutrition and physical activity targets.”)

**2.4. Self-monitoring of outcomes of behaviour & 7.1. Prompts/cues** (“Continuation of email, text and phone reminders to self-monitor weight, diet and physical activity, adjusted for maintenance time frame”)

**3.1 Social support (unspecified)** (Specific “relapse” weight loss program if weight rebounds by more than 3% of their baseline weight, including an initial phone call from a trained consultant.)

**10.10. Reward outcome** (“Congratulation emails for successful maintenance of lost weight.”)

**Coughlin, 2013; Brantley, 2008; Funk, 2010; Steven, 2008**

**WLM Website BFS**

**1.1 Goal setting behaviour:** [tailored] “physical activity goal for Phase II was to increase moderate-intensity physical activity to an average of at least 45min per day at least five times each week” “The website provided personally tailored goal setting”

**2.3 Self monitoring** **behaviour** (“food and Fitness diaries with web entry of weight, minutes of exercise, total daily calories and number of food and fitness diaries kept, at least weekly”)

**2.2 Feedback on behaviour and 2.4 feedback on outcome** (“Printable progress reports and graphs of self-reported data, tailored welcome messages, reminder prompts (e-mail and phone) based on website usage.” “automated feedback in the form of weight and exercise graphs, personalized messages, and tailored progress reports to assist with motivation and relapse prevention”)

**7.1 Prompts/cues** (“Continuously available, encourage weekly contacts, welcome messages and reminders to return to the website if needed [email]; Reminders to return if needed [interactive voice]. Prompts to return to website if needed [personal phone calls])

**1.2 Problem Solving (mode of delivery - web-based system** (Website - “customizable action plans from interactive relapse prevention, self-management modules”; “Customizable action plans from interactive problem-solving, self-management modules)

**1.4 Action planning** **[mode of delivery - in person]** ( “participants developed a personal action plan regarding weight, physical activity, and dietary goals”)

**1.4 Action planning** **[mode of delivery - web-based system]** ( “customizable action plans from interactive relapse prevention, self-management modules”; Orientation visit - “participants developed a personal action plan regarding weight, physical activity, and dietary goals”)

**3.1 Social support** (“Bulletin board”; “bulletin board for communication with other participants”)

**5.1 Information about health consequences** (“Available on the website”; “links to reliable health and weight loss related information, and printable program materials and resource”) (“weekly tips and polls”)

**9.1 credible source (**“Available on the website”; “links to reliable health and weight loss related information...”)

**Theory Background**

“...behaviorally-based theories such as social cognitive theory [44], behavioral self-management techniques [45], the transtheoretical model [46,47],and motivational enhancement techniques [48,49]”

**Tailoring**

“The website provided personally tailored goal setting and problem-solving programs to help participants develop action plans for perceived obstacles to success. In order to tailor the intervention to the needs of the participant, the action plan could be updated anytime.”

“automated feedback in the form of weight and exercise graphs, personalized messages, and tailored progress reports to assist with motivation and relapse prevention”

**Mode of Delivery**

demonstrating how to use the website. During this orientation, participants developed a personal action plan regarding weight, physical activity, and dietary goals using the interactive website.” … “ invited to attend a group reorientation visit following a 12 month data collection. The purpose of this visit was to familiarize the participants with new features on the website, and re energize their maintenance efforts”

Personal Contact Group (same content, differs in mode of delivery) “Behavioral counseling usingMotivational Interviewing,delivered through personal phone calls and in-person visits”; Goal setting behaviour: [tailored] “All participants were provided with individualized goals for caloric intake based on personalized needs for weight maintenance or additional weight loss.”

“physical activity goal for Phase II was to increase moderate-intensity physical activity to an average of at least 45min per day at least five times each week”

**Espel-Huynh et al, 2019 - Refresher course**

**1.1. Goal setting behaviour & 8.7 graded tasks** (“In the introductory session, patients are instructed to gradually increase physical activity beyond that achieved during weight loss. If a patient has not yet reached the program's initial goal of 150 min/week, they are encouraged to continue increasing toward that point. Patients at or above 150 min/week gradually work toward 200 and ideally 250 min/ week for the duration of the maintenance period.)

**1.2. Problem solving** (“Each refresher course focuses on a new topic and introduces new behavioral challenges specific to longer-term maintenance. Examples include coping with the potential boredom of routine diet and physical activity patterns, managing cravings and urges, and connecting daily weight management efforts with one's broader personal values (e.g., health or family).”)

**1.3. Goal setting outcome** (“A 5-lb. weight loss goal is also prescribed for each 4-week period.”)

**1.9 Commitment** (“Commitment to weight maintenance and personal values”)

**2.3. Self-monitoring of behaviour & 2.4 Self-monitoring of outcomes of behaviour** (“During the Refresher Courses in months 7 and 10, patients are instructed to self-monitor their weight, caloric intake, and physical activity every day.”)

**2.7. Feedback on outcomes of behaviour (**“At the end of each week during the Refresher course, automated feedback is provided to patients, similar in content to that provided during the initial 3-month weight loss period.”

Small weight gains (relative to starting maintenance weight) are addressed through a color- coded self-regulation program [17]. Weight loss maintenance or loss falls in the “Green/GO” zone. Patients in the “Yellow/CAUTION” zone (1–3 lbs. gained) are asked to make small dietary changes to reduce intake until reaching the Green zone again. Patients who reach the “Red/STOP” zone (≥ 4 lbs. gained) resume full weight loss efforts until returning to maintenance goal weight.”)

**5.1 Information about health consequences** (“Understanding metabolism and increasing lifestyle physical activity”)

**Evans et al., 2015; Sniehotta et al., 2019 - NuLevel**

**1.1 Goal setting (Behaviour) [diet and PA]** (Identify and formulate a SMART physical activity goal for WLM(e.g. daily step count)

**1.2 Problem Solving [Relapse prevention* as discriminated by the original authors]** (Explain lapse/ relapse distinction and importance of problem solving; Specifying potential sources of social support, barriers, and possible solutions)

**1.2 Problem Solving [Barrier identification* as discriminated by the original authors]** (Identify situations in which participant struggles to be active**;** identify possible barriers, and possible solutions; Discuss possible obstacles to implementing the plan, and possible solutions to apply)

**1.2 Problem Solving (Coping planning* as discriminated by the original authors)** (Participant generates “ if ... then ...” formulations using a volitional help sheet (Armitage 2014) physically drawing a line to link situations with several possible solutions)

**1.2 Problem solving [MoD - SMS]** [red zone, participants confirm wish to lose weight] (“You’ve lost weight before, so you know a lot about which methods work for you and which don’t. With that in mind, do you currently have a plan for how you’d like to lose weight? Alternatively, would you like our help in developing one? Please text back to let us know.”)

**1.3 Goal setting (outcome) [weight] (**Agree overall weight goal and regain thresholds for red and yellow zones)

**1.4 Action planning [diet and PA]** (Discuss how, where, when and with whom to put the plan into place)

**1.5 Review behavioural goals [diet and PA - MoD face to face]** (goal review/resetting process)

**1.5 Review behavioural goals** **[MoD - SMS; when goal not met in one week; when goals met for 3 consecutive weeks]** (“You have mastered your activity goals congratulations! Why not set yourself some new goals for next week, since you’ve mastered these ones? If so, follow this link to write a new plan (Link). If not, you need to do nothing.”)

**1.7 Review of outcome goal** (“Thanks for telling us you’d prefer to avoid further weight gain, but don’t wish to lose weight at the moment. We are here to support and encourage you every step of the way. In this case, we reset your yellow zone weight to put you back into the green zone, then provide usual weight maintenance support. Are you happy for us to do this?”)

**2.2 Feedback on behaviour [diet and PA - MoD face to face]** (Review and discuss current physical activity)

**2.2 Feedback on behaviour [MoD - SMS]** (“...and behavioural goals (dietary and physical activity) in response to the information they enter each week”) / “thanks for submitting your activity and eating goal diary entries - they help you keep on track to meet your weight goal”)

**2.3 Self-monitoring of behaviour [weekly; MoD - SMS]** (“please complete your diary today, by following this link”)

**2.3 Self-monitoring of behaviour [diet and PA; MoD Face to Face]** (Discuss eating goal self-monitoring using online study interface. Explain input of eating data to online ‘diary’, SMS feedback and goal review/resetting process)

**2.4 Self-monitoring of outcome [weight; MoD SMS]**  (“Tailored SMS feedback allows them to evaluate their progress towards their outcome goal(weight)”...) [as required pre-defined] (“Don’t forget to weigh yourself today - it’s important for maintaining your weight loss)

**2.4 Self-monitoring of outcome [weight; MoD Face to Face]** (Demonstrate and encourage weight self-monitoring using online study interface; explain weight-related SMS feedback system)

**2.7 Feedback on outcome [MoD - Website]** (“NULevel intervention participants are able to see the weights sent by their SIM-enabled scales displayed on a graph using the online platform.)

**2.7 Feedback on outcome [weekly/as required pre-defined; MoD - SMS]** (“this week you managed to stick to your green zone weight: good job.”)

**2.7 Feedback on outcome [MoD Face to Face]** (“Provide feedback on performance”)

**3.1 Social support (unspecified)** (“On request of the participant, individual telephone calls with a member of the research team can be scheduled to discuss specific problems with WLM”)

**3.2 Social support** [on participant request] (“We are glad to hear that you’d like our help in developing a plan to get your weight back on track. This is done most easily with a phone call: please text back CALL ME if you’d like a call, with a suitable day and time. Please text NO CALL if you don’t want us to ring: we’ll continue to support your plan development by SMS”)

**4.1 Instruction on how to perform the behaviour [PA, tempting situations]** (Provide instruction on how to perform the behaviour and information on where/when to perform the behaviour)

**4.1 Instruction on how to perform the behaviour [for social support and self-management] (**Explain weekly online diary contains questions on WLM confidence; health, wellbeing and priority placed on WLM in previous week; Explain options to contact the research team (byemail, SMS and diary tick-box))

**5.1 Information about health consequences [PA]** (Highlight importance of physical activity to WLM and overall health, and discuss ways to become more active, if desired)

**7.1 Prompts/cues** [every six weeks] (Have you looked at your weight graph recently? If not, simply log in at {link: login} and click ‘weight’ to view your progress. Log in details not working or forgotten? Please text back and we’ll help!”)

**10.4 Social reward** [when goals met for 3 consecutive weeks] (“You have mastered your activity goals congratulations!) (“You’re back in the green zone - congratulations for getting your weight back on track!”)

**12.2 Restructuring the social environment [diet and PA]** (Plan social support/social change; specify potential sources of social support)

**12.3 Avoidance/reducing exposure to cues for the behaviour** [non specified, date based, eg. Christmas] (“Many people decide to throw dietary caution to the wind on Christmas Day–don’t worry, we won’t try to dissuade you! It’s only one day of the year. However, if you fancy a delicious, indulgent Christmas Day that’s (surprisingly) under 2500calories, then look no further: {link: web content}”).

**12.5 Adding objects to the environment** (“every participant (intervention and control arm) receives a wirelessly”)

**15.3 Focus on past success** (Review weight history, overall trajectory and number of WL attempts)

**Theory Background**

**Self-Regulation Theory:** ”Effective behavioural principles”; It uses technology to facilitate the monitoring of weight, behavioural goals and risk factors for lapses and it provides feedback and reinforcement. As such it is based on effective behavioural principles [15, 26]. This approach also allows tailoring of intervention components to the participants’ progress”.

**Tailoring**

“This approach also allows tailoring of intervention components to the participabts progress.”

“NULEVEL scale levels of support based on traffic light system”

**Gerber et al., 2013**

**1.2. Problem solving & 7.1. Prompts/cues** (“This channel included videos of the instructor (reminders, motivational talks and problem solving ideas); cooking and grocery shopping; narrative stories from participants recorded during the weight loss maintenance phase; and other programmes (e.g. SisterTalk, a health programme for African-American women).[6]”)

**3.1. Social support unspecified** (“Women in the intervention arm received monthly telephone support calls during the weight maintenance phase (average duration 10–12 min).”)

**4.1. Instruction on how to perform a behaviour & 6.1. Demonstration of the behavior**

(“The other two channels included exercise videos (beginning and advanced), such as “Sit and Be Fit” and “Walk Away the Pounds”.”)

**12.1. Restructuring the physical environment & 12.5. Adding objects to the environment (**“they received DVRs with wireless adapters or wired connections for Internet access. A technician was available for home visit installation and instruction. As the recorders were typically connected to living room television sets, the videos could be viewed at routine television watching times. In addition, efforts were made to arrange space near the televisions for home exercise.”)

**Leahey et al., 2016**

**1.1. Goal setting behaviour & 1.3. Goal setting outcomes [mode of delivery - group session]** (“During the group session, participants also completed a goal setting activity in which they specified their weekly weight, calorie, and activity goals depending on whether they wanted to continue to lose weight or maintain”)

**1.1. Goal setting behaviour [mode of delivery - email]** (“Specifically, every two weeks, participants were emailed a new behavior to monitor (e.g., Weeks 1–2: pedometer steps; Weeks 3–4: “red foods”) and given goals for each behavior (e.g., 10,000 steps/ day; limit “red foods” to 1 serving/day).”)

**1.2. Problem solving** (“and brainstormed problem solving solutions in the event that they did not meet their goals.”)

**1.4. Action planning** (“developed a plan to achieve the goals”)

**2.3. Self-monitoring of behaviour & 2.4. Self-monitoring of outcomes of behaviour** (“Each week participants self-monitored their weight and the prescribed diet or activity behavior for at least 5 days, and emailed the self-monitoring data to their professional coach (a registered dietitian with training in behavioral weight control)

**4.1. Instructions on how to perform behaviour** (“CB Pro participants also attended one group session during which they were oriented to their program and encouraged to engage in core weight maintenance strategies as noted above. After this one-hour session, CB Pro participants also received their entire intervention via email.”)

**7.1. Prompts/cues** (“Weekly reminders to submit self-monitoring information were framed using regret aversion lan- guage (“Don't miss out on your money, be sure to submit your information by Sunday at midnight”).”

**10.1 Material incentive (behaviour)** (Participants were informed that they would receive between $1–$10/week for submitting their records, but the specific schedule of reinforcers was not provided to participants in advance.”)

**10.2. Material reward (behaviour)** (“In addition, each week that participants self-monitored and submitted ≥5 days of weight and diet or activity information to their coach, they received a monetary reinforcement.)

**10.4 Social Reward** (“Given that self-monitoring is consistently associated with better maintenance outcomes, (Butryn et al., 2007; Wing and Hill, 2001) both the social and financial reinforcers were provided contingent on self-monitoring.” ; “If they did not email the information, no social reinforcement or contact from the coach was provided.” ; “Each week participants self-monitored their weight and the prescribed diet or activity behavior for at least 5 days, and emailed the self-monitoring data to their professional coach (a registered dietitian with training in behavioral weight control), they received an email from their coach providing social reinforcement (support, encouragement).)

**10.10. Reward outcome** (“Weight maintenance was also incentivized. Those who maintained their weight loss in full at their assessment session received $25. Thus, maximum potential earnings were $185/participant, provided at the assessment visit.”)

**14.6. Situation-specific reward** (“Given that self-monitoring is consistently associated with better maintenance outcomes, (Butryn et al., 2007; Wing and Hill, 2001) both the social and financial reinforcers were provided contingent on self-monitoring.”

“If they did not email the information, no social reinforcement or contact from the coach was provided.”)

**Nakata et al., 2019**

**1.1. Goal setting behaviour [Mode of delivery web-based system]** (The default step count and MVPA targets per day were set at 8000 steps and 20 min, respectively; however, the personal targets were set individually with a planned gradual increase (500–2000 steps/day and 5–20 min/day). One author (Y.N.) checked the participants' body weight and physical activity using the web-based system”)

**1.1. Goal setting behaviour [Mode of delivery - group session]** (“which involved changes in diet and exercise. Specifically, the recommendations included an energy-restricted diet of 1200 and 1600 kcal/day for women and men, respectively; as well as a minimum physical activity increase of 1000 kcal/week”)

**2.2. Feedback on behaviour & 2.7. Feedback on outcomes of behaviour** (“The web-based system automatically created two graphs of the participants' activity patterns, one showing changes in body weight and step count (upper left in Fig. 1) and another displaying a scatter diagram consisting of step count (X axis) and MVPA (Y axis; upper right in Fig. 1) (Aoyagi and Shephard, 2013).”

“One author (Y.N.) checked the participants' body weight and physical activity using the web-based system and provided monthly personalized feedback (approximately 350–450 Japanese characters, corresponding to 200–250 words in English) for 24 months.”

**2.3. Self-monitoring of behaviour & 2.4. Self-monitoring of outcomes of behaviour** (“participants in the web-support group were instructed to measure their body weight daily and to wear the activity monitor during waking hours”

**3.1. Social support unspecified** (“We provided textbooks, notebooks and group-based support sessions during weeks 1, 2, 3, 4, 6, 8, 10, and 12.”)

**3.1. Social support unspecified** (“Typical advices comprised of monthly evaluation of weight and physical activity, applause and encouragement.”)

**8.7 Graded tasks** (“however, the personal targets were set individually with a planned gradual increase (500–2000 steps/day and 5–20 min/day).”)

**12.5 Adding objects to the environment** (“The web-support group was provided with a weight scale (BC-569; Tanita, Tokyo, Japan) and an activity monitor (Kenz Lifecorder GS; Suzuken, Nagoya, Japan), and received intervention via a web-based, weight-loss maintenance program (Fig. 1).”)

**Scott et al., 2019 - NoHoW Trial**

**2.3 Self-monitoring of behaviour & 2.4 Self-monitoring of outcome**

(“The active control arm includes a toolkit dashboard containing weight, steps and sleep graphs using data from the Fitbit devices, healthy eating and mood data (self-scored on a five-point scale).

**2.7 Feedback on outcome of behaviour** (“Individualised feedback automatically (...) provided by the toolkit in response to the individual’s weight trajectory. Feedback is generated from the data extracted from three sources: (1) weight from the Fitbit Aria scales; (2) activity and sleep from the Fitbit Charge 2; and (3) user engagement with the toolkit, including day of the week. After daily usage patterns are analysed, feedback is displayed in the toolkit consisting of one a set of short statements (eg, “your weight management appears better when you are more active”)

**7.1 Prompts/cues** (“The toolkit sends automated weekly emails to participants to encourage engagement, prompt log-in and recommends sessions to complete (arms 2–4 only) (...) If no activity is detected after 21, 28 and 35 days).

**12.5 Adding objects to the environment** (“Participants were asked to wear the Fitbit Charge 2 at all times and to weigh themselves ≥twice weekly. All arms include instructions for self-weighing and use of activity trackers”)

**Theory Background**

“Guidance for complex interventions was followed during the toolkit development specifying the intervention logic models and theory-driven behaviour change techniques, which will be detailed in a separate publication.”

**Tailoring**

“Individualised feedback automatically (...) provided by the toolkit in response to the individual’s weight trajectory. Feedback is generated from the data extracted from three sources: (1) weight from the Fitbit Aria scales; (2) activity and sleep from the Fitbit Charge 2; and (3) user engagement with the toolkit, including day of the week. After daily usage patterns are analysed, feedback is displayed in the toolkit consisting of one a set of short statements (eg, “your weight management appears better when you are more active”).

**Mode of Delivery**

“consisting of videos/audios, interactive graphs, text, images, questionnaires and mini-apps.”; “weekly arm-specific emails are sent during the active 6-month intervention phase, which provides WLM information, suggestions and reminders to visit specific content.”.

**Thomas et al., 2011**

**2.4. Self-monitoring of outcomes of behaviour & 7.1. Prompts/cues** (Participants were sent a monthly e-mail requesting a report of their current weight.)

**4.1. Instruction on how to perform the behaviour**  (weekly e-mail from the dietitian called ‘Tip of the Week’. Tips gave dietary, behavioural and exercise advice)

**Wing et al., 2008**

**1.2. Problem solving & 10.10. Reward outcome** (“and were provided with monthly token reinforcers if they were within 1.4 kg of starting weight. If weight gains of 1.4 – 2.2 kg were experienced, participants were taught to problem solve, and for weight gains ≥ 2.3 kg to restart weight loss efforts. Those who gained ≥ 2.3 kg were also offered additional counseling (via email for Internet group and in person or by phone for face-to-face group). Lessons were presented by the same staff across the two conditions either in face-to-face classes or Internet chat rooms. The lessons focused on issues related to maintenance of weight loss and recommended strategies that had been used successfully by NWCR members to maintain their weight loss, including exercising 60 minutes per day.”)

**2.4. Self-monitoring of outcomes of behaviour** (“Participants submitted their weight weekly (via phone or web-based form)”; “taught to weigh themselves daily and to use the information from the scale to determine if changes in eating and exercise behaviors were needed”)

**3.1 Social support (unspecified) (**Those who gained ≥ 2.3 kg were also offered additional counseling.)

**3.2 Social support (practical)** (Lessons were presented by the same staff across the two conditions either in face-to-face classes or Internet chat rooms**)**

**4.1. Instructions on how to perform behaviour** (“Both groups were taught to weigh themselves daily and to use the information from the scale to determine if changes in eating and exercise behaviors were needed. “)

**Tailoring**

Strategies delivered based on weight maintenance (green) or weight gain (yellow/red)

**Mechanisms of Action**

**Brindal et al., 2016**

**[Behavioural Regulation]**

Multiple studies have reported on the benefits of weight monitoring for weight control [24,25].

**[Emotion] & [Perceived susceptibility/vulnerability]**

Well-being monitoring was included to allow people to track their moods, so that they can see any link between emotional states and other behaviors (in summary sections) but also to allow a point of EMI for the coping behavioral support tool .Previous apps have tried to capture this style of processes successfully without persuasive features [27]. Mood states included were designed to capture pleasant and unpleasant moods as well as moods with high versus low arousal [28].

**[Behavioural Regulation] & [Goals] & [Feedback Processes]**

Given consistent evidence about the link between diet, exercise and weight loss maintenance [2], the app includes tools to monitor these behaviors. The aim is to design an approach that is generic enough to support a variety of different approaches for diet and exercise regimes that individuals had adopted in their weight loss journey. Therefore, national recommendations were used to provide feedback and set targets [30,31].

**[Emotion] & [Optimism] & [Skills] & [Motivation] & [Needs]**

Previous studies have reported that supportive features can be beneficial for mood amongst women losing weight [19]. While some factors (eg, goal setting, motivation, and self-efficacy) may promote adherence to weight loss programs, psychological factors, such as stress and depression may inhibit peoples’ ability to maintain weight losses[32-34]. Daily motivational messages were constructed based on inspirational quotes and thoughts to inspire optimism and the potential for higher resilience and problem-solving ability[14]. In accordance with HAPA theory, and as Figure 1A shows, these messages transition from a focus on general motivation and action planning through to coping planning.

**[Emotion] & [Optimism] & [Beliefs about capabilities]**

Psycho-education contained within the information buttons provides guidance on maintaining positive mental well-being based on positive psychology and cognitive therapy. This component is designed to promote self-awareness and equip people with greater self-efficacy to recognize and modify their behaviors in order to maintain their positive behavior changes.The coping workshopping interface only appears once a large change in mood or stress is detected from the data entered. Inorder to capture information about both potential triggers and coping resources, the interface appears for both negative changes in mood or stress.

**[Skills] & [Beliefs about capabilities] & [Self-image]**

Focusing on the coping resources that users have successfully applied rather than simply how they could fix hassles is an important behavioral element in the effort to assist users in building effective coping strategies.

**Collins et al., 2010; Collins et al., 2019**

**[Behavioural regulation] & [Environmental Context and Resources & Social Influences]**

Achieving and maintaining weight loss requires behaviour change. Bandura’s Social Cognitive Theory [14] proposes that behaviour change is influenced by environmental factors, personal factors, and attributes of the behaviour itself. This interaction is referred to as ‘reciprocal determinism’, as each factor may affect or be affected by the others.

**[Behavioural Regulation] & [Goals] & [Skills]**

The online program targeted key mediators including self-efficacy (e.g. goal setting, self-monitoring of weight, body measurements, exer- cise and diet),

**[Beliefs about Consequences & Knowledge]**

outcome expectations (e.g. knowledge- based web components),

**[Social Learning/Imitation]**

modelling (e.g., interactive website features and demonstrations)

**[Social Influences]**

and social support (i.e. forums, blogs, feedback, email and/or tele- phone contact).

**Coughlin, 2013; Brantley, 2008; Funk, 2010; Steven, 2008**

**WLM Website BFS**

**[Skills] & [Behavioural Regulation] & [Reinforcement]**

Reinforce existing behavioral self-management strategies

Facilitate and encourage new self-management skills

**[Beliefs about capabilities]**

Improve self-efficacy for long-term weight management

**[Environmental Context and Resources]**

Remain fresh and inviting to encourage regular, long-term contact

**[Social Influence]**

Promote social support among website users

**Espel-Huynh et al., 2019**

No MoAs reported

**Evans et al., 2015; Sniehotta et al., 2019 - NuLevel**

**[Knowledge]**

-correct any existing misperceptions about a healthy and sustainable diet

**[Goals]**

-establish clear behavioural goals, action plans and coping plans

**[Self-Efficacy]**

-to enhance self-efficacy for successful weight management and relapse prevention

**[Behavioural regulation]**

-increase the rate of self-weighing, self-monitoring of behaviour, and the use of self-regulation strategies

**[Behavioural Cueing]**

-Prompting participants to develop routines of monitoring progress on weight, physical activity and eating behaviour goals is hypothesised to be a key change mechanism in weight management

**[Memory, Attention and Decision process]**

- thereby conserving their psychological resources and avoiding intervention fatigue and ego depletion

**[Motivation & Self-image & Values]**

- support participants in focusing on motives which have been hypothesised to facilitate maintenance such as enjoyment of WLM behaviours [47], identity coherent with healthy lifestyle choices [46], self-determination [48], and satisfaction with weight outcomes [41]

**[Behavioural regulation & Skills & Memory, Attention and Decision process] & [Environmental Context and Resources & Social Influences & Goals]**

- Text messages also facilitate individual self-regulation [15,43] and the development of healthy habits and routines [46]; they support the management of personal resources [34,49], social and environmental challenges/opportunities [37], and goal conflicts and priorities [50].

**[Feedback processes] & [Behavioural regulation] & [Needs] & [Skills] & [Belief about Capabilities]**

- Additional support in the yellow and red zone is hypothesised to help solving problems, managing temporary lapses and preventing relapses [32] as well as to provide social support [51]. - Steps 1–4 are hypothesised to lead to healthier eating patterns [52], higher levels of physical activity and therefore reduced weight regain compared to controls. The maintenance of weight loss as well as the experience of control over one’sbody weight are hypothesised to result in higher quality of life [53].

**Gerber et al., 2013**

No MoAs reported

**Leahey et al., 2016**

**[Belief about consequences]**

Components designed to address the high cost–benefit ratio thought to undermine weight loss maintenance (Jeffery et al., 2000, 2004; Foreyt et al., 1981; Perri et al.,1987).

**[Reinforcement] & [Social Influences]**

Specifically, to increase “benefits,” or rewards, for weight management, two universal reinforcers were used: social reinforcement and monetary reinforcement.

**[Environmental Context and Resources]**

To decrease boredom or “costs” of long-term adherence, the maintenance program included a variety of different evidence-based strategies for weight loss maintenance, and the specific behavioral strategy changed every two weeks. Each of these approaches is detailed below.

**[Environmental Context and Resources]**

To reduce behavioral “costs” for engaging in weight management behaviors, behavioral variety was used (Jeffery et al., 2009; Epstein

This approach was used to mitigate boredom and thus effort.

**[Behavioural Regulation & Reinforcement]**

Given that self-monitoring is consistently associated with better maintenance outcomes, (Butryn et al., 2007; Wing and Hill, 2001) both the social and financial reinforcers were provided contingent on self-monitoring.

**Nakata et al., 2019**

No MoAs reported

**Scott et al., 2019**

**NoHoW**

**[Behavioural Regulation] & [Emotion] & [Motivation]**

...thematic modules targeting self-regulation and motivation (arm 2), emotion regulation (arm 3) and combined self-regulation and emotion regulation (arm 4).

**Thomas et al., 2011**

No MoAs reported

**Wing et al., 2008**

**[Behavioural regulation]**

-The interventions were based on self-regulation theory.
